# Pan-cancer identification of clinically relevant genomic subtypes using outcome-weighted integrative clustering

**DOI:** 10.1186/s13073-020-00804-8

**Published:** 2020-12-03

**Authors:** Arshi Arora, Adam B. Olshen, Venkatraman E. Seshan, Ronglai Shen

**Affiliations:** 1 Department of Epidemiology and Biostatistics, Memorial Sloan Kettering Cancer Center, New York, NY USA; 2 Department of Epidemiology and Biostatistics, University of California at San Francisco, San Francisco, CA USA; 3 Helen Diller Family Comprehensive Cancer Center, University of California at San Francisco, San Francisco, CA USA

**Keywords:** Integrative clustering, Supervised learning, Patient survival, Prognostic molecular stratification

## Abstract

**Background:**

Comprehensive molecular profiling has revealed somatic variations in cancer at genomic, epigenomic, transcriptomic, and proteomic levels. The accumulating data has shown clearly that molecular phenotypes of cancer are complex and influenced by a multitude of factors. Conventional unsupervised clustering applied to a large patient population is inevitably driven by the dominant variation from major factors such as cell-of-origin or histology. Translation of these data into clinical relevance requires more effective extraction of information directly associated with patient outcome.

**Methods:**

Drawing from ideas in supervised text classification, we developed *survClust*, an outcome-weighted clustering algorithm for integrative molecular stratification focusing on patient survival. *survClust* was performed on 18 cancer types across multiple data modalities including somatic mutation, DNA copy number, DNA methylation, and mRNA, miRNA, and protein expression from the Cancer Genome Atlas study to identify novel prognostic subtypes.

**Results:**

Our analysis identified the prognostic role of high tumor mutation burden with concurrently high CD8 T cell immune marker expression and the aggressive clinical behavior associated with *CDKN2A* deletion across cancer types. Visualization of somatic alterations, at a genome-wide scale (total mutation burden, mutational signature, fraction genome altered) and at the individual gene level, using *circomap* further revealed indolent versus aggressive subgroups in a pan-cancer setting.

**Conclusions:**

Our analysis has revealed prognostic molecular subtypes not previously identified by unsupervised clustering. The algorithm and tools we developed have direct utility toward patient stratification based on tumor genomics to inform clinical decision-making. The *survClust* software tool is available at https://github.com/arorarshi/survClust.

## Background

Cancer is a complex disease with heterogeneous clinical outcomes. Understanding how patients respond to treatment and what drives disease progression and metastasis is critical for managing and curing the disease. Linking comprehensive molecular profiling data with patient outcome carries great promise in addressing such important clinical questions. This requires innovative statistical and computational methods designed for integrative analysis of multidimensional data sets to model intra-tumor and inter-patient heterogeneity at genomic, epigenetic, and transcriptomic levels. Each of these molecular dimensions is correlated yet characterizes the disease in its own unique way. In order to arrive at a comprehensive molecular portrait of the tumor, multiple groups have proposed statistical and computational algorithms to synthesize various channels of information including methods developed by us (iCluster [1, 2]) and others (PARADIGM [3], CoCA [4], SNF [5], CIMLR [6]) to stratify disease populations. However, the majority of the work has focused on unsupervised clustering that utilizes the molecular data alone.

A complexity of applying unsupervised learning to molecular phenotypes lies in that it does not necessarily lead to unique answers. This limitation is well understood in the field of text learning. Consider the problem of clustering a collection of documents where multiple data substructures can be present including authorship, topic, and style. The outcome of the clustering is likely driven by a mixture of these underlying structures. As a result, there is often no single “right” answer in unsupervised clustering problems. In most complex data applications, many local optima exist that poses special challenges in optimization. Xing et al. [7] proposed a weighted distance metric allowing users to specify what they consider “meaningful” in defining similarity toward a more efficient and local optima-free clustering performance.

Drawing analogy with the text learning problem described above, the molecular profile of a tumor is influenced by a multitude of factors including cell-of-origin [8], histology (e.g., squamous vs. adenocarcinoma), tumor microenvironment (e.g., immune cell infiltration [9]), dedifferentiation states [10], and specific pathway activation [11]. Conventional unsupervised clustering applied to the most variable features is inevitably driven by the dominant variation from major factors, for example, cell-of-origin [8] or ancestry [12] (germline variation) in the study cohort. When patient outcome-related stratification is of interest, a more directed clustering approach is needed.

To overcome the current limitation of molecular clustering analysis, we developed the *survClust* algorithm as a supervised learning approach that aims to identify cancer subtypes that are not just molecularly distinct but also prognostically significant. It is an outcome-weighted integrative clustering algorithm for survival stratification based on multidimensional omics-profiling data. The algorithm learns a weighted distance matrix that downweights molecular features with no relevance to the outcome of interest. This method can be used on individual platforms alone, or by integrating various molecular platforms, to mine biological information leading to distinct survival subgroups. In this study, we analyzed over 6000 tumors across 18 cancer types. Each disease type was classified by *survClust* based on six molecular assays—somatic point mutations, DNA copy number, DNA methylation, mRNA expression, miRNA expression, protein expression, and the integration of the six assays. The results have revealed novel survival subtypes not previously identified by unsupervised clustering.

## Methods

### Data source

The analysis in this study was conducted on the Cancer Genome Atlas dataset. This included six molecular data types: somatic point mutations, DNA copy number, DNA methylation, mRNA expression, miRNA expression, and protein expression across 6209 tumor samples covering 18 cancer types. Data pre-processing and normalization procedures are described in Additional file 1: Supplementary Note.

### survClust workflow

Let ***X***_***m***_ be the *m*th (*m* = 1,…,*M*) data type of dimension *N*_*m*_ (number of samples in the *m*th data type) by *p*_*m*_ (number of features in the corresponding data type). Rows are samples and columns are molecular features. Data types may consist of continuous (gene expression, copy number log-ratio, DNA methylation, miRNA, protein expression) or binary (mutation status) data. Overall survival is defined as the time from diagnosis to death or last follow-up. The number of samples in each tumor type and the number of molecular features in each data type are summarized in Additional file 1: Table S1.

For a pair of two samples *a* and *b*, the weighted distance [7] is calculated as follows:

1 $$ {d}_w\left(\boldsymbol{a},\boldsymbol{b}\right)=\sqrt{{\left(\boldsymbol{a}-\boldsymbol{b}\right)}^{\boldsymbol{T}}\ \boldsymbol{W}\left(\boldsymbol{a}-\boldsymbol{b}\right)}, $$

where ***a*** and ***b*** are feature vectors of length *p* for samples *a* and *b,* respectively, and ***W*** is a *p* × *p* diagonal weight matrix with ***W*** = *diag* {*w*_1_, …, *w*_*p*_}. Samples are close to each other when the value of *d*_*w*_ is small and dissimilar when *d*_*w*_ is large.

The weights *w*_*j*_ (*j* = 1, …, *p*) are obtained by fitting a univariable Cox proportional hazards model fitted for each feature:

2 $$ h\left(t|{\boldsymbol{x}}_{\boldsymbol{p}}\right)={h}_o\times \exp \left({\boldsymbol{x}}_{\boldsymbol{j}}^{\boldsymbol{T}}\ast \beta \right), $$

where *t* represents the survival time, ***x***_***j***_ is the *j*th column of matrix ***X*** of length *N*, *h*_0_ is the baseline hazard function, *β* is the regression coefficient, and exp(*β*) is the hazard ratio (HR).

We consider the absolute value of HR on the logarithmic scale as the weight *w*. An HR = 1 corresponds to the null that the feature is not associated with survival. This is reflected in a log (1) = 0 weighting in the distance matrix. Since ***W*** is a diagonal matrix with diagonal elements *w*_*j*_ (*j* = 1, …, *p*), we can simply use Euclidean distance for computing distances if we transform the data as follows:

3 $$ {\boldsymbol{X}}^{\prime }=\boldsymbol{X}\ast {\boldsymbol{W}}^{\frac{\mathbf{1}}{\mathbf{2}}}. $$

Euclidean distances are sensitive to scale of the observations so after incorporating weights, we standardize the data by its grand total:

$$ \frac{{\boldsymbol{X}}^{\prime }}{\sum \limits_{\boldsymbol{i}}\sum \limits_{\boldsymbol{j}}{x}_{ij}^{\prime }}, $$

where $\sum \limits_{\boldsymbol{i}}\sum \limits_{\boldsymbol{j}}{x}_{ij}^{\prime }$ is the grand total of the weighted matrix ***X***′, with *i* rows (*N* samples) and *j* columns (*p* features). Then, one can compute the pairwise distance between samples *a* (*i* = 1) and *b*(*i* = 2) as:

$$ {d}_w\left(\boldsymbol{a}^{\prime },\boldsymbol{b}^{\prime}\right)={d}_w\left(\boldsymbol{b}^{\prime },\boldsymbol{a}^{\prime}\right)=\sqrt{\sum \limits_{j=1}^p{\left({a_j}^{\prime }-{b_j}^{\prime}\right)}^2.} $$

Conversely, a weighted distance matrix ***D*** is calculated for all pairwise samples across *M* data types. All samples having full survival information are kept, and the union of all samples (*N*_union_) across *M* data types is utilized when analyzing a wide number of samples. Non-overlapping samples in data types are added as *NA* to have a uniform set of *N*_union_ samples.

The integrated weighted distance matrix is calculated by averaging over the weighted distance matrices:

4 $$ {\boldsymbol{I}}_{\boldsymbol{w}}=\sum \limits_{m=1}^M{\boldsymbol{\gamma}}_{\boldsymbol{m}}{\boldsymbol{D}}_{\boldsymbol{m}}, $$

where ${\boldsymbol{\gamma}}_{\boldsymbol{m}}=\frac{\mathbf{1}}{\boldsymbol{M}}\boldsymbol{\forall}\boldsymbol{m}.$ The integrated weighted matrix ***I***_***w***_ averages the inter- and intra-sample similarity profiles over the *M* data types. ***I***_***w***_ is then processed by *survClust* via classical multidimensional scaling (MDS) [13] and clustered using k-means [14]. Classical MDS assumes Euclidean distances; however, in cases of non-Euclidean distances, Mardia et al. [15] provided a method to obtain the resulting positive semidefinite scalar product matrix. Note that *I*_*w*_ follows the Euclidean norm and hence can be represented in *n* − 1 dimensions. The strong assumption of the Euclidean norm is also important for k-means, as it is essentially trying to assign samples to the closest centroid or calculating the sum of squared deviations from centroids.

### Weighted distance metric for mutation data

Somatic mutation data is represented as a binary data matrix where each entry is coded as 1 if the *j*th gene is mutated in the *i*th sample, and 0 otherwise. A challenge with the mutation data matrix is the sparsity. It is known that somatic mutation data exhibit a long-tailed distribution in which a relatively small number of variants appear in tumors frequently while the vast majority of variants occur extremely infrequently. We consider genes that are mutated in > 1% of the samples. After incorporating weights, this data is no longer binary, but it still remains sparse. Due to such data sparsity, computing the Euclidean distance is not appropriate and may lead to inflated distance measures [16]. To combat this problem, we propose a weighted binary distance metric for such a scenario.

Let ${\boldsymbol{X}}_{\boldsymbol{mut}}^{\prime }$ be the weighted mutation data matrix (see Eq. 3) of dimension *N* (samples) by *p* (genes). Then, the pairwise distance between sample vectors ***a*** and ***b*** is calculated as follows:

$$ {d}_w\left(\boldsymbol{a},\boldsymbol{b}\right)={d}_w\left(\boldsymbol{b},\boldsymbol{a}\right)=\frac{w_{01}+{w}_{10}\ }{w_{01}+{w}_{10}+{w}_{11}}, $$

where

*w*_01_ = sum of weights of *p* features that are zero in sample vector ***a*** but non-zero in sample vector ***b*;**

*w*_10_ = sum of weights of *p* features that are non-zero in sample vector ***a*** but zero in sample vector ***b*;**

*w*_11_ = sum of weights of *p* features that are non-zero in sample vector ***a*** and non-zero in sample vector ***b*.**

Note that *d*_*w*_(***a***, ***b***) is a proportion of the sum of effect sizes in which only one is non-zero among those in which at least one is non-zero [17].

### Cross-validation

*survClust* classifies sample populations by incorporating outcome information. Cross-validation was used to prevent overfitting and arrive at more generalizable solutions. The *cv.survclust* function performs cross-validation for the desired number of folds and outputs cross-validated solution labels. In the data analysis, we performed 5-fold cross-validation in the following steps: (1) Split the data into 5 random partitions, label 4 of them as the training sets and the remaining one as the test set. (2) The weighted distance matrix was calculated from the training data set alone (Eq. 1). *survClust* clustering was performed to arrive at outcome-weighted labels in the training set. (3) Each test set sample was assigned a class label based on its molecular feature vector and weights derived from the training set. We note the survival information for the test sample was not used in assigning its class label, ensuring an unbiased assessment of survival association for the class assignment on the test set. (4) Step 2 was repeated until predictions were made on all 5 test data sets across all 5 folds. (6) Clusters were tracked by *centroid relabeling* (Additional file 1: Supplementary Note) across folds, and we obtained outcome-weighted class labels for our entire dataset. This concluded one round of cross-validation. All results shown here are results from cross-validated labels across 50 rounds of cross-validation. Cluster labeling consistency was preserved across rounds of cross-validation via a similar approach to centroid relabeling. The final class label for a sample was assigned based on a consensus voting, i.e., the class that the sample was assigned the highest number of times in the 50 rounds of cross-validation. This is achieved by another function called *consensus.summary*.

### Choice of the number of clusters ***k***

We use both the logrank test statistic and a standardized pooled within-cluster sum of squares calculated from cross-validation to choose an appropriate *k*.

### The logrank statistic

The logrank test statistic is based on a non-parametric approach that quantifies survival difference between resulting subtypes and makes no assumption about the survival distributions. It tests the null hypothesis that there is no difference in survival between the groups.

For a particular *k* cluster solution, we have *k* cross-validated labels. Each class is distinct in survival, and we can compare the difference between classes using the logrank test statistic as follows [18]:

$$ {\chi}^2=\frac{\sum \limits_k\left({O}_k-{E}_k\right)}{\sqrt{V}}, $$

where *O*_*k*_ = observed number of events in the *k*th group over time, *E*_*k*_ = expected number of events in the *k*th group over time and *V* = ∑ *Var* (*O*_*k*_ − *E*_*k*_) = ∑ *V*_*k*_.

For a two-group comparison, the logrank statistic follows a chi-square distribution with 1 degree of freedom. A value greater than 3.84 is considered statistically significant at an alpha of 0.05. The optimal *k* is the one with the maximum logrank statistic.

### Standardized pooled within-cluster sum of squares

Here we calculate the pooled within-cluster sum of squares and standardize it by the total sum of squares similar to methodology used in the gap statistic [19] to select the appropriate number of clusters.

Suppose that the final labels have clustered the data into *k* clusters *C*_1_, *C*_2_, …. *C*_*k*_, with *C*_*r*_ denoting the indices of observations in cluster *r*, and *n*_*r*_ = |*C*_*r*_|. Let

$$ {w}_r=\sum \limits_{\begin{array}{c}i,j\in {C}_r\\ {}i>j\ \end{array}}{I_w}_{ij}, $$

where *w*_*r*_ is the sum of all pairwise distances in cluster *r*, {*ij*} represents a pair of samples belonging to a cluster *C*_*r*_, and ***I***_***w***_ is calculated from Eq. 4. Then, the standardized pooled within-cluster sum of squares is:

$$ {W}_s=\sum \limits_{r=1}^k{w}_r/\sum \limits_{\begin{array}{c}i\\ {}i>j\end{array}}\sum \limits_j{I_w}_{ij}. $$

Here *W*_*s*_ decreases monotonically as the number of clusters *k* increases. The optimal number of clusters is where *W*_*s*_ is minimized and creates an “elbow” or a point of inflection, where addition of more clusters does not improve cluster separation. Another property of *W*_*s*_ is that it can be used to compare among different datasets as it lies between 0 and 1 after standardization. This is useful for comparing *survClust* runs between individual data types and when we integrate them.

### Adjusted Rand index

The Rand index is used to measure agreement between two classification labels. When this Rand index is adjusted for chance, it is called the adjusted Rand index. The Rand index and adjusted Rand index have a maximum of 1 and a minimum of 0. Here 0 means the two data labels have no shared information and 1 means they are the same labels.

### Simulation

**Fig. 1:**
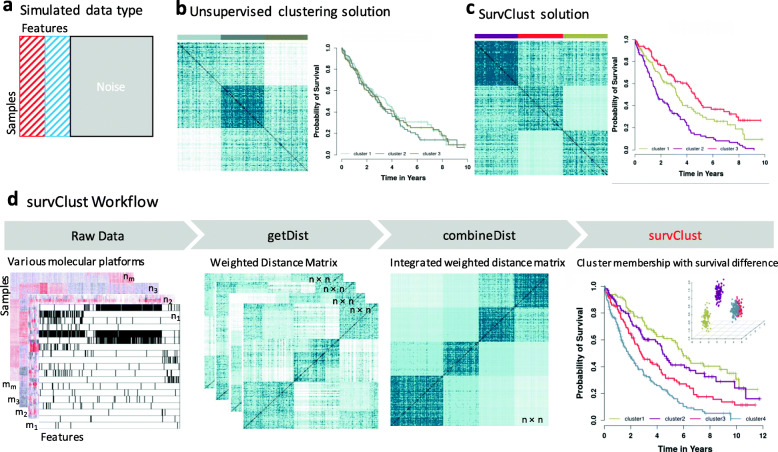
Overview of *survClust.* **a** A simulated data example, consisting of features that define 3 patient subtypes without direct association with survival (shaded in red), features that define 3 patient subtypes with distinct survival outcome (shaded in blue), and random features generated from Gaussian noise (gray). **b** Euclidean distance matrix demonstrating patient-level pairwise similarity, with darker blue shade representative of higher similarity. Color panels above the distance matrix show the three-class solution obtained by unsupervised algorithm via k-means and the concordance between the simulated 3 survival subtypes (the truth). Kaplan-Meier curves for the 3 unsupervised subtypes show no distinction in survival outcome. **c** *survClust* employs a patient outcome-weighted distance matrix to identify the desired subtypes with distinct Kaplan-Meier curves. **d** *survClust* allows integrative analysis of multiple data modalities to identify survival-associated molecular subtypes

Continuing from the simulation study presented in Fig. 1, we go into detail about cross-validation and how to choose **k** for a *survClust* run. In Fig. 1, the input matrix was subjected to 50 rounds of 3-fold cross-validation (2/3 training and 1/3 test). The *survClust* fit for a cluster ***k*** based on training data from each fold was used to predict cluster membership for the test data. Final sample labels were aggregated over all folds and cluster meaning was preserved across folds via centroid relabeling (see Additional file 1: Supplementary Note).

The logrank test statistic and standardized pooled within-cluster sum of squares was calculated for the consolidated test labels over 3-folds for each round. Additional file 1**:** Fig. S1c summarizes these metrics for 50 rounds of cross-validation for **k** = 2–7. The figure shows that logrank is maximized for *k* = 3, and the standardized pooled within-cluster sum of squares has an elbow at *k* = 3, pointing to the optimal *k* of 3. The final class labels are assigned by consolidating solutions across all folds in all rounds of cross-validations.

It is interesting that the choice of *k* is strongly echoed across these two different metrics. Another simulation scenario is presented in Additional file 1: Fig. S2. Here survClust was able to identify the 4-class simulated truth when there is conflicting information present in individual platforms.

## Results

### The *survClust* model: an overview

The molecular profile of a tumor often harbors information on a multitude of factors including cell lineage, tumor microenvironment, cell differentiation, and other clinical and histopathological features. Some of these factors are associated with treatment response and/or survival outcome, while others are not. If a particular patient outcome (e.g., patient survival) is of interest, a more supervised approach is needed. We demonstrate this using a simulated data example (Fig. 1a, Additional file 1: Fig. S1). In this scenario, we simulated three risk subgroups in a cohort of 300 hypothetical patient samples with distinct survival hazard rates in each subgroup (a median survival of 4, 3, and 2 years, respectively). A set of 15 features was then simulated from a mixture Gaussian distribution with different means in the three risk subgroups. Another set of 15 features was simulated in the same way but permutated to disrupt the feature-risk group association. A third group of 270 features was simulated from Gaussian noise. Figure 1b shows that an unsupervised clustering using the K-means algorithm failed to identify the survival subtypes in the context of complex feature variations. To identify outcome-associated clustering solution, *survClust* utilizes a weighted distance metric:

$$ d\left(\boldsymbol{a},\boldsymbol{b}\right)=\sqrt{{\left(\boldsymbol{a}-\boldsymbol{b}\right)}^T\ \boldsymbol{W}\left(\boldsymbol{a}-\boldsymbol{b}\right)}, $$

where (***a***, ***b***) denote a pair of sample vectors measured for *p* features and ***W*** is a diagonal weight matrix over *p* features with ***W*** = *diag* {*w*_1_, …, *w*_*p*_}. The weights *w*_*p*_ ′ *s* are obtained by fitting a univariable Cox proportional hazards model for each feature in the training data with repeated training-test sample splits for cross-validation (see more details in the “Methods” section). Figure 1c shows that *survClust* was able to identify the true risk groups with 97.15% accuracy [95% CI = 94–100%], whereas the accuracy from an unsupervised clustering was 67.50% without reducing the effect of noise features and features unrelated to survival.

Our algorithm allows the integration of multiple data modalities. Given *m* data types measured over the respective feature space (Fig. 1d), the algorithm learns a weighted distance matrix from each molecular data type by incorporating a vector of Cox regression hazard ratio as weights. Each feature is weighed and a pairwise distance matrix is calculated **(**we refer to this step as **getDist)**. This step reduces the computation considerably by transforming the problem from sample by feature to sample by sample. Note that different sample sizes across data types are allowed, i.e., a sample can be measured for some but not all platforms. Next, the weighted pairwise distance matrices are integrated by summing overweighted *m* data types (**combineDist**), which retains all samples with at least one data type available, with complete pairwise information. **survClust** then projects the integrated and weighted distance matrix into a lower dimensional space via multidimensional scaling (MDS) and then clusters sample points into subgroups via the K-means algorithm. More details can be found in the “Methods” section.

### *survClust* is more powerful than unsupervised clustering in identifying clinically relevant molecular subtypes

We applied *survClust* to the TCGA data set including 6209 tumor samples in 18 cancer types to identify survival outcome-associated subtypes defined by somatic mutation, DNA copy number, DNA methylation, mRNA expression, and protein expression, individually and integratively. A summary of the sample sizes and feature space is included in Additional file 1: Table S1. Additional file 1: Table S2 compares the survival association (logrank statistic) for the *survClust* integrated subtypes versus those derived from unsupervised clustering methods commonly used in TCGA studies including COCA and iCluster. The logrank statistic compares estimates of the hazard functions of each subgroup to the expected values under the null hypothesis (all subgroups have identical hazard functions). Larger logrank statistic suggests stronger evidence of survival association. For example, although unsupervised classification of the liver cancer samples elaborates on molecularly distinct subtypes, they do not exhibit significant survival separation (*P* = 0.42, logrank statistic = 1.71) [20]. The *survClust* integrative classification, on the other hand, identified subtypes that are molecularly distinct and also show significant survival difference (*P* < 0.001, logrank statistic = 21.56) (Additional file 1: Table S2, Fig. S8). In addition to the multi-platform integrative analysis, we also present a comprehensive comparison of the *survClust* classification vs TCGA unsupervised clustering analysis for each individual molecular platform as summarized in Additional file 1: Table S3–7. By differentially weighting the molecular features by the corresponding survival association in constructing the distance matrix, we show that *survClust* is more powerful for identifying subtypes that are directly relevant to stratify the outcome of interest, leading to substantially more distinct survival subgroups than those existing molecular subclasses obtained by unsupervised clustering. To further demonstrate, we highlight the *survClust* analysis of low-grade glioma and kidney papillary renal cell carcinoma below.

### *survClust* identifies a poor prognostic *IDH-*mutant low-grade glioma subgroup

Low-grade gliomas (LGG) have a unique molecular footprint, characterized by *IDH1/2* mutation status and codeletion in chromosome 1p and19q regions of the genome [21]. As shown previously, mutations in *IDH1* and *IDH2* genes are present in a majority of the low-grade gliomas and define a subtype associated with favorable prognosis [22]. *IDH*-mutant tumors with chromosome 1p and 19q codeletion (*IDH*-mutant-codel) exhibit the most prolonged survival times followed by *IDH*-mutant tumors without the codeletion (*IDH-*mutant-non-codel), with *IDH-*WT tumors demonstrating more aggressive clinical behavior. We performed *survClust* on 6 available molecular platforms (somatic mutation, DNA copy number, DNA methylation, mRNA expression, and protein expression) in 512 LGG samples as profiled by the TCGA. The optimal number of clusters *k* was chosen by assessing *survClust* fits over logrank test statistics and standardized pooled within-cluster sum of squares in cross-validation (see the “Methods” section). Cross-validation was performed to ensure unbiased estimation of survival association and to avoid overfitting.

**Fig. 2:**
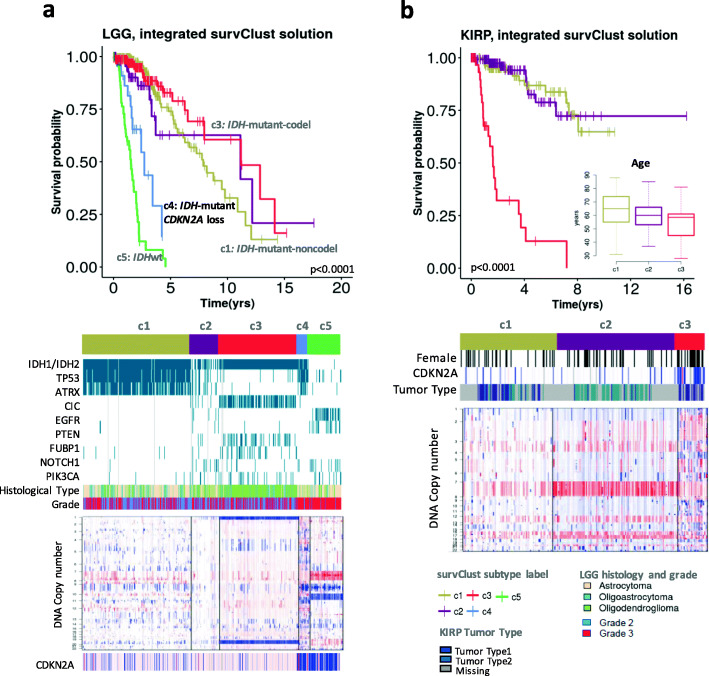
Outcome-weighted integrative clustering of low-grade glioma (LGG) and kidney papillary cell carcinoma using *survClust*. **a** *survClust* identifies an *IDH*-mutant *CDKN2A*-loss subtype similar to *IDH*-wt tumors in terms of the aggressive clinical behavior. Top: Kaplan-Meier curves of the integrated *survClust* subtypes of LGG. Middle: *Panelmap* summarizing major association of mutational and clinical features of the integrated LGG subtypes. Bottom: Copy number profile for each of the integrated subtypes. **b** *survClust* identifies prognostic kidney papillary renal cell carcinoma (KIRP) subtypes

The integrated *survClust* solution for LGG was optimized at *k* = 5, with the *IDH*-mutant-codel (c3) and *IDH*-mutant-non-codel (c1) subtypes associated with good prognosis as expected (Fig. 2a). By contrast, the *IDH*-WT subclass (c5) showed association with poor survival, enriched for mutations in *EGFR* and *PTEN* gene and concurrent chromosome 7 gain and 10 loss, resembling glioblastomas. Interestingly, *survClust* identified a small *IDH*-mutant subtype characterized by *CDKN2A* deletion (c4) that showed markedly worse survival among the *IDH*-mutant tumors, similar to the *IDH*-WT group (c5) that tends to behave far more aggressively with prognosis similar to glioblastomas. In addition, a copy number quiet subgroup (c2) was identified that showed high expression of mir-1307 and mir-29c (Additional file 1: Fig. S3). These results highlight the strength of *survClust* in identifying clinically relevant molecular stratifications and the potential to refine the existing paradigm in glioma subtyping to inform clinical decision-making.

### *survClust* identifies prognostic subtypes of kidney papillary renal cell carcinoma (KIRP)

Three survival distinct subtypes were identified using *survClust* integrating DNA copy number, mRNA expression, DNA methylation, and miRNA and protein expression profiles of 289 tumor samples. The c3 subtype was associated with poor survival (median survival time = 1.63 years) (Fig. 2b), younger age (median age 57 years), and the female gender (55%). The defining genomic characteristics include *CDKN2A* loss and arm-level gains in multiple chromosomes including 7, 12, 15, and 17 as described previously [23].

### *survClust* identifies clinically relevant mutational subgroups across cancer types

**Fig. 3:**
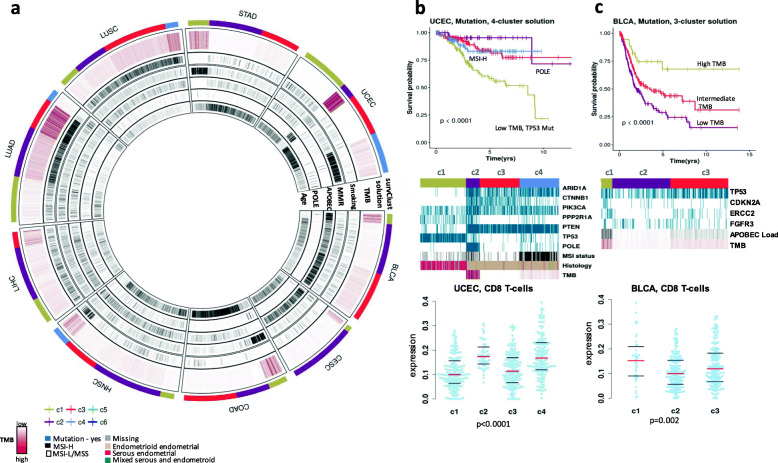
*survClust* identifies mutational subtypes associated with survival across cancer types. **a** *Circomap* showing total mutation burden (TMB) in brown color and mutational signatures (smoking, MMR, APOBEC, POLE, and aging) in tumors across bladder (BLCA), cervical (CESC), colon (COAD), head and neck (HNSC), liver (LIHC), lung adenocarcinoma (LUAD), lung squamous cell (LUSC), stomach (STAD), and endometrial (UCEC) cancers. Outer circle indicates mutation-based *survClust* membership. **b** *survClust* mutation subtypes in endometrial cancer**.** From top to bottom*:* Kaplan-Meier curves for the 4 mutation subtypes; *panelmap* depicting significantly mutated genes, MSI status, histology, and TMB associated with the subtypes; and beeswarm plot showing CD8 T cell marker expression (*y*-axis) across the 4 subtypes (*x*-axis). Red line depicts the median, and top and bottom black bars represent the 25th and 75th percentile, respectively. **c** *survClust* mutation subtypes in bladder cancer. From top to bottom: Kaplan-Meier curves for the 3 mutation subtypes; *panelmap* depicting significantly mutated genes, APOBEC load, and TMB associated with the 3 subtypes; and beeswarm plot showing CD8 T cell expression (*y*-axis) across the 3 subtypes (*x*-axis)

*survClust* is a flexible framework and can be applied to individual data types for patient stratification. For example, somatic mutation-based stratification is often of interest in a clinical sequencing setting. To illustrate this, we applied *survClust* to mutation data alone using a hazard ratio weighted binary distance-based clustering. A *circomap* plot was created to facilitate annotation and visualization of the results across cancer types (Fig. 3a). *survClust* identified high TMB subgroups in nearly all cancer types included in this analysis. Correlating mutational signatures [24] with these subtypes in *circomap* further revealed etiology underlying these hypermutated tumors. The smoking signature tracks lung cancer (LUSC and LUAD) and the subset of head and neck cancer (HNSC) with elevated TMB. The DNA mismatch repair (MMR) signature tracks high TMB subgroups in stomach cancer (STAD), endometrial cancer (UCEC), and colon cancer (COAD). The APOBEC signature is prevalent in the bladder (BLCA) and cervical cancers (CESC). Finally, the aristolochic acid signature (signature 22) is enriched in a liver cancer subgroup identified by *survClust* (Additional file 1: Fig. S4e), which is consistent with aristolochic acid and their derivatives being implicated in liver cancers in Asian populations [25].

In endometrial cancer, *survClust* confirmed a previously known ultra-high mutated subtype associated with the POLE mutation signature (c2) and a hypermutated microsatellite instability (MSI) (c4) subtype [26] (Fig. 3b). The *panelmap* in Fig. 3b (middle panel) shows that c4 correlated well with clinical MSI status (*P* < 0.001) and predominantly carried mutants in *ARID1A*, *PIK3CA,* and *PTEN* genes. The c1 subtype, consisting of primarily high-grade serious tumors, was associated with the worse outcome with a 5-year survival of 58% compared to 95%, 84%, and 83% for c2 (POLE), c3, and c4 (MMR), respectively, and characterized by higher frequency of mutations in *TP53*, *PPP2R1A* genes, low TMB, and older patients with serous endometrial tumors (60%). The c3 subtype was characterized by a higher frequency of *CTNNB1* mutants. Immune cell decomposition data derived using the CIBERSORT [27] algorithm was also correlated with the subgroups. Interestingly, high expression of CD8 T cell immune marker was observed in the POLE (c2) and MSI (c4) subtype (*P* < 0.001) (Fig. 3b).

*survClust* stratified the bladder cancer cohort into 3 TMB subgroups—with high (c1), intermediate (c3), and low (c2) mutation burden. The c1 subtype was associated with good outcome, high TMB, high neo-antigen load, high APOBEC load, and high expression of the CD8 T cell immune marker (*P* = 0.002) (Fig. 3c). The c3 subtype showed intermediate TMB and APOBEC load with a median survival time of 3.48 years. Patients with a low TMB and low APOBEC load performed the worst in terms of survival with a median survival time of 1.91 years.

A similar pattern emerged when *survClust* was run on colorectal cancer mutation data classifying the disease population into three clusters—two low TMB groups and a MMR-associated high TMB group (c1) (Additional file 1: Fig. S4b). c1 was also associated with CD8 T cell infiltration (*P* = 0.004) and showed concordance with MLH1 silencing status. A similar subdivision of the low TMB group by *TP53* mutation status was seen where c3 carried *TP53* mutant samples unlike c2. Correlation with histology revealed significant enrichment of the mucinous adenocarcinoma subtype in c1 and c2 (c1, *n* = 20, 29%; c2, *n* = 24, 20%) compared to c3 (*n* = 9, 5%). In addition to the hypermutated subtypes of endometrial, bladder, and colorectal cancers, we also observed high TMB subgroups with concurrently high expression of CD8 T cell markers in the cervical cancer c1 subtype (Additional file 1: Fig. S4a and S5a), head and neck cancer c4 subtype (Additional file 1: Fig. S4c, S5c), lung adenocarcinoma c3 subtype (Additional file 1: Fig. S4f and S5f), lung squamous cell carcinoma c4 subtype (Additional file 1: Fig. S4g and S5g), and stomach cancer c1 subtype (Additional file 1: Fig. S4h and S5h). There are prior observations that high mutational burden is associated with increased neo-antigen load and activated T cell infiltration in lung cancer [28]. Our analysis revealed that such associations may be more widely present in multiple cancer types.

### *survClust* identifies distinct copy number subtypes associated with clinical features across cancer types

**Fig. 4:**
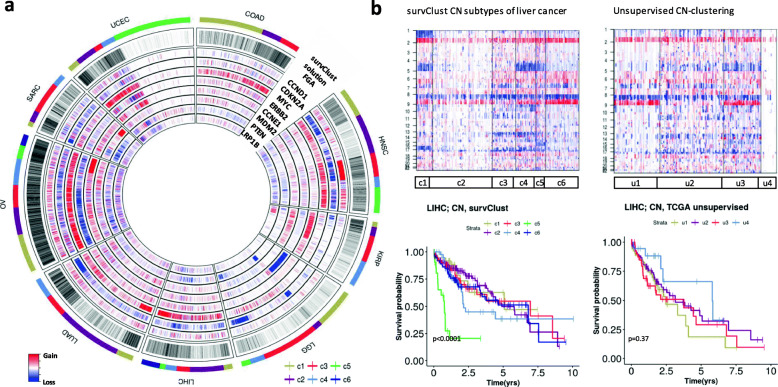
*survClust* identifies copy number patterns associated with patient survival outcome across various cancer types. **a** *Circomap* showing fraction genome altered (FGA) and gene-level copy number alterations in each tumor across colorectal *(*COAD), head and neck (HNSC), kidney renal papillary cell carcinoma (KIRP), low-grade glioma (LGG), liver (LIHC), lung adenocarcinoma (LUAD), ovarian (OV), soft tissue sarcoma (SARC), and endometiral (UCEC) cancers. The outer circle indicates the *survClust* membership. **b** *survClust* is more powerful than unsupervised clustering in identifying survival-associated copy number subtypes in liver cancer

To identify copy number alterations that define clinically relevant subtypes, segmented data of 18 cancer types was processed via the CBS algorithm [29] and analyzed with *survClust*. Subtypes characterized by different degrees in the Fraction of Genome Altered (FGA) emerged in various cancer types (Fig. 4). Interestingly, low FGA was associated with better survival in several cancer types including colon, head and neck, lung adenocarcinoma, soft tissue sarcoma, and endometrial cancer (Additional file 1: Fig. S6 and S7).

The *circomap* plot in Fig. 4a also revealed associations of subtypes with high-level amplification of major cancer genes including *CCND1* amplification in head and neck cancer (c3), *CCNE1* (c5) and *AKT2*(c6) amplification in ovarian cancer, and *MDM2* amplification (c4) in sarcoma (S Additional file 1: Fig. S6). Notably, amplification of the 19q13.2 region in the ovarian cancer c6 subtype harboring the *AKT2* gene is associated with poor survival (Additional file 1: Fig. S7f, Table S8) which was consistent with previous findings that *AKT2* amplification is associated with ovarian cancer aggressiveness [30]. The *CCND1* amplified subtype of head and neck cancer (c3) was also associated with poor survival (Additional file 1: Fig. S7b). Amplification in the *MYC* gene is broadly present in multiple cancer types (Fig. 3a *circomap*). Among cancer gene deletions, *CDKN2A* loss defined multiple subgroups associated with poor survival including papillary kidney cancer (c1), low-grade glioma (c4), lung adenocarcinoma (c4), and soft tissue sarcoma (c1) (Additional file 1: Fig. S6 and S7).

Colorectal cancer was classified into three varying FGA subtypes with prognostic implications. c1 had low FGA, while c2 and c3 carried heavy genome alterations (Additional file 1: Fig. S6a). Even though c1 and c2 had dissimilar FGA, they performed similarly in terms of survival as compared to c3, which had poor outcome with median survival time of 4.5 years (Additional file 1: Fig. S7a). Gain in the MYC gene was seen throughout the cancer type, and c2 was uniquely characterized by loss of the chromosome 20 p-arm, which harbors the hsa-mir-103–2 previously reported to be downregulated in colorectal tumors [31, 32].

*survClust* is designed to capture the contribution of survival-associated molecular features and reduce the influence from those that are not related to the outcome of interest. Figure 4b provides another example that this approach is better at identifying prognostically relevant subtypes compared to the unsupervised clustering approach applied in the original study [20]. *survClust* identified 6 unique CN groups in liver cancer with significant survival differences among subgroups. The c5 subtype was characterized by high FGA and associated with poor outcome with a median survival time of 0.77 years. This cluster was distinguished by a loss of chromosome 15. The c2 subtype was associated with the lowest FGA and a median survival time of 6.81 years. The c4 subtype was enriched for *CDKN2A* deletion with a median survival time of 2.15 years. In contrast, unsupervised clustering generated subgroups with distinct molecular differences but did not show any separation in terms of survival.

### Integration of multiple data types enhances the identification of survival distinct subgroups

**Fig. 5:**
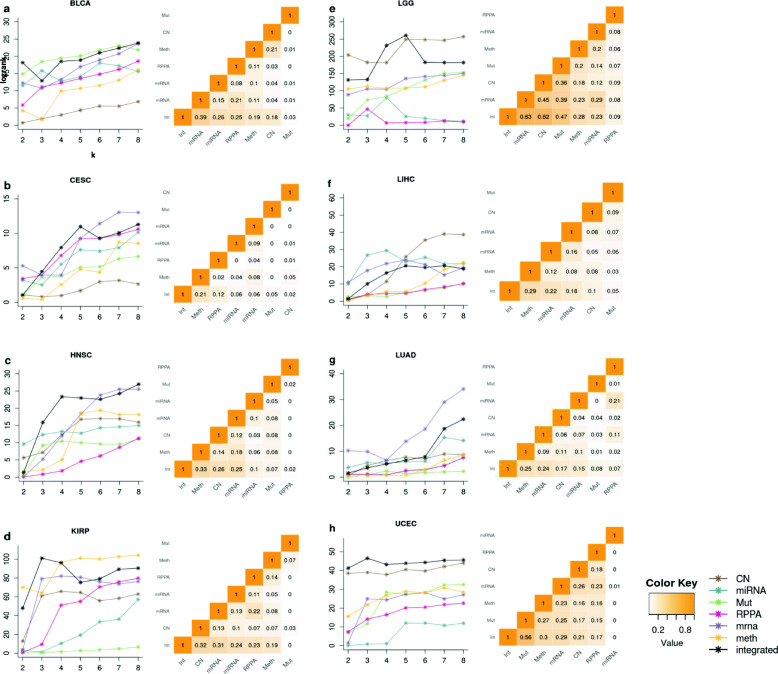
Integration of multiple data types enhances the identification of survival distinct subgroups. **a–h** Each panel has two plots: the plot on the left summarizes median cross-validated logrank statistic across *k* = 2 to 8 (number of clusters). Each line is a data type (see legend), and the black line represents the *survClust* run on integrating all 6 platforms. Plot on the right summarizes the adjusted Rand index between cross-validated *survClust* solutions of individual data types and the integration of all. In this comparison, the *survClust* solution was chosen for an appropriate *k* which maximized logrank statistic and minimized the standardized pooled within sum of squares

Figure 5 shows that the integrated *survClust* solution outperformed individual platforms based on the cross-validated logrank statistics for multiple cancer types including cervical cancer, head and neck cancer, papillary kidney cancer, lower grade glioma, and liver and endometrial cancers. In general, the integrated solutions always emerge at or near the top in performance as compared to the individual platform-specific solutions.

Next, we used the adjusted Rand index (RI) to evaluate the concordance between different solutions. RI is calculated as the proportion of sample pairs that are assigned together in the same cluster in one solution versus another, adjusted for what is expected by random chance. It provides an indirect measure of how much a particular data type contributes to the integrated solution. A non-zero adjusted RI across solutions would suggest shared biology across assay types in some tumors. For example, the mutation subtypes of endometrial cancer (Fig. 5h) have the highest adjusted RI (0.56) as compared to the integrated solution, which is consistent with the fact that POLE and MSI are the two major prognostic subtypes that are predominantly defined through mutation burden (Fig. 3b). Nevertheless, the integrated solution also shows clearly that there is additional information in DNA methylation, DNA copy number, and mRNA expression being effectively incorporated by *survClust* that improves the survival stratification. In bladder cancer, the integrated solution is most concordant with the mRNA cluster solution (adjusted RI = 0.39), which indicates influence by mRNA features toward integration (Fig. 5a). Classification by mutation data type seemed to have little or no overlap between other assays (adjusted RI close to 0), although the integrated solution retained some information. (adjusted RI = 0.03).

The integrated solution classified cervical cancer samples better than the rest of the platforms and pointed toward a 5-cluster solution (Fig. 5b). Interestingly, a high degree of heterogeneity among different platforms was observed as represented by a small adjusted RI across the board. The head and neck cancer integrated solution showed great improvement over individual platforms for *k* > 2 solutions. The *k* = 4 integrated solution clearly resulted from effective integration of multiple data types including DNA methylation, DNA copy number, and mRNA expression with adjusted RIs of 0.33, 0.26, and 0.25, respectively (Fig. 5c). In this case, RPPA provided very little information toward the integrated solution.

The integrated survClust analysis stratified papillary kidney cancer type into 3 groups, with CN sharing maximum information with the integrated solution (adjusted RI = 0.32), followed by mRNA (0.31), miRNA (0.24), RPPA (0.23), and methylation (0.19). Lower grade glioma displayed a wide range of variability among platform type in terms of the logrank statistic (logrank statistic, *x*-axis from 0 to 250). The *k* = 5 integrated solution performed the best among the 6 platforms with larger contributions from mRNA (RI = 0.63), copy number (RI = 0.62), and mutation (RI = 0.57) (Fig. 5e). The integrated solution of liver cancer did not show much improvement over individual assay types. Note that we did not use protein data while integrating as more than half was missing (RPPA, *n* = 182; integrated *n* = 371). miRNA, mRNA, and copy number showed high median logrank statistics over rounds of cross-validation demonstrating their role as potential prognostic classifiers.

## Discussion

Cancer is a complex disease. Integrative analysis of multi-omic molecular profiling has the potential to unpack the complexity of the disease to reveal insights into disease etiology and to identify subtypes with distinct outcome for clinical utility. Unsupervised clustering methods have been developed to define cancer subtypes across multiple data modalities [1–4] and to stratify cancer patients into molecularly distinct subtypes. In some cases, these molecular subtypes were shown to be associated with survival such as the integrated subtypes of breast cancer [33]. In other cases, unsupervised molecular stratification led to subtypes related to other factors such as the etiologically distinct molecular subtypes in gastric adenocarcinoma cancer [34], the histology-associated molecular subtypes of esophageal cancer [35], and the cell-of-origin-driven pan-cancer subtypes [8].

When unsupervised clustering does not lead to survival-associated subtypes, a supervised approach is needed in order to identify clinically relevant patient stratifications. Nevertheless, there is a lack of supervised clustering methods specifically designed for cancer genomics application, which emphasizes the potential utility of *survClust*. A strong use-case scenario for such an approach is explained in the simulation example presented in Fig. 1, where unsupervised clustering alone was not enough to distinguish survival-associated clusters unless survival unrelated features are downweighed, by an approach like *survClust*. Similar limitation of such a canonical clustering approach can also be seen in Fig. 4, where classic unsupervised clustering identified copy number distinct classes not associated with any prognostic significance in liver cancer samples.

Our approach is innovative in several aspects. First, it directly incorporates survival information in the clustering approach by utilizing weighted distance matrices. Specifically, molecular features are weighted by their corresponding log-hazard ratio (logHR) estimated from univariable Cox regression from training data. As a result, informative features will have large weights in clustering whereas noninformative features will have weights close to zero and thus minimal influence on the clustering. When unsupervised clustering fails to identify survival-associated subtypes, we show that our supervised analysis allows effective extraction of survival information and leads to clinically relevant molecular stratification. Secondly, *survClust* facilitates multi-modal clustering by integrating the weighted distance matrices. It then projects the integrated and weighted distance matrix into a lower dimensional space via multidimensional scaling (MDS) in which sample points are organized into subgroups via the K-means algorithm. Our analysis showed that the integrated analysis outperformed the individual data types in multiple cancer types, highlighting the importance of the multi-omic approach. Finally, we developed various visualization tools including *panelmap* and *circomap* that greatly facilitate the interpretation of the results.

As more clinically annotated genomic data becomes available as a result of clinical sequencing programs [36, 37], our method will provide a useful tool to facilitate patient stratification for clinical decision-making. In this study, we analyzed 18 cancer types in ~ 6200 tumors. Each disease type was classified by *survClust* based on six molecular assays—somatic point mutation, DNA copy number, DNA methylation, mRNA expression, miRNA expression, protein expression, and integration of the aforementioned six assays.

The supervised clustering approach provides a more direct way to identify survival-associated molecular subclasses, leading to substantially more distinct survival subgroups than those existing molecular subclasses obtained by unsupervised clustering. In this study, *survClust* analysis of copy number data alone identified aggressive clinical behavior of tumors with *CDKN2A* deletion in multiple cancer types, and poor survival for a subtype of ovarian cancer carrying aberration on Chromosome 19. Furthermore, the integrated clustering analysis via *survClust* shed light on clinical subtypes that were not identified by individual platform alone. For instance, the integrative analysis of the glioma using *survClust* revealed a small novel subtype characterized by *CDKN2A* deletion and *IDH1/2* mutation (Fig. 2a), whereas the individual platform analysis mostly agreed with previously known subtypes (Additional file 1: Fig. S4d, S6d). This again underlines the power of the proposed approach in identifying novel clinically relevant subtypes by integrating across ‘omics data types and their underlying association with outcome.

There are several limitations to our current approach. First, the current method focuses on time to event outcome (patient survival) as the clinical endpoint of interest. A future extension of the algorithm will be needed for molecular stratification associated with other types of outcome such as treatment response which requires a modification of the weighting scheme appropriate for binary and categorical types of outcome. Secondly, the current integration across multiple data types does not allow different weights for the distance matrices computed from each individual data modality. A weighted matrix integration may further allow more flexible integration of the different data types. Also, the integrated results presented in the paper were run on a large feature space (Additional file 1: Table S1) that retains as many samples as possible across individual data types. Although such an approach is more comprehensive, the computational cost is high. Feature selection approach may be considered to further improve computational efficiency in future work.

## Conclusions

In molecular stratification analysis, it is common to apply an existing unsupervised clustering method followed by a post hoc clinical association analysis. Such a “two-step” approach does not always guarantee the molecular subtypes are prognostically distinct as we demonstrated in our study. To address this challenge, we developed the *survClust* algorithm as a more powerful supervised learning approach, aiming at the identification of cancer subtypes that are not just molecularly distinct, but also prognostically significant. We analyzed over 6000 tumors across 18 cancer types from the Cancer Genome Atlas study, across six molecular data types including somatic point mutations, DNA copy number, DNA methylation, mRNA expression, miRNA expression, protein expression, and the integration of the six data modalities. The results have revealed prognostic molecular subtypes not previously identified by unsupervised clustering.

## Availability and requirements

Project name: survClust

Project home page: https://github.com/arorarshi/survClust

Operating system(s): Linux, Mac OS X, Windows

Programming language: R

License: GNU, GPL

Any restrictions to use by non-academics: None

## Supplementary Information

**Additional file 1:** Supplemental File containing all Supplemental Figures, Supplemental Tables, and Supplemental Note including additional details on the method and data analysis. **Table S1.** Supplementary Table summarizing input data of 18 cancer types across number of samples analyzed and total number of features that went into clustering for mutation, Copy Number (CN), Methylation, mRNA expression, miRNA and Protein. **Table S2.** Supplementary Table showing comparisons of survClust integrated solution versus unsupervised clustering results from published TCGA studies. **Table S3.** Summary of survival association for the survClust Copy Number solutions versus unsupervised TCGA solutions using the logrank statistic. **Table S4.** Summary of survival association for the survClust Methylation expression solution versus available TCGA solutions using the logrank statistic. **Table S5.** Summary of survival association for the survClust mRNA expression solution versus available TCGA solutions using the logrank statistic. **Table S6.** Summary of survival association for the survClust miRNA expression solution versus available TCGA solutions using the logrank statistic. **Table S7.** Summary of survival association for the survClust Protein expression solution vs available TCGA solutions using the logrank statistic. **Table S8.** Cross tabulation of the ovarian cancer copy number survClust class labels vs *AKT* gene Copy number changes. **Figure S1.** survClust simulation analysis. **Figure S2.** A second simulation example. **Figure S3.** survClust integrated solution of low grade glioma (LGG). **Figure S4.** survClust identifies tumor mutation burden (TMB) patterns across cancer types. **Figure S5.** CD8 expression stratified by the survClust mutation subclasses across various cancer types. **Figure S6.** survClus tidentifies copy number alteration patterns across cancer types, Global view of Copy Number Changes. **Figure S7.** survClust identifies FGA patterns across cancer types that associate with survival (Kaplan Meier curves). **Figure S8.** survClust integrated solution across six molecular platforms in various cancer types. **Supplementary Note** Description of data pre-processing, simulation details and the centroid re-labeling approach.

## Data Availability

*survClust* is freely available as an R package at our github repository [38]. Mutation data along with relevant clinical annotations were plotted using *panelmap* [39]. The *Circos* package was used to make pan-cancer plots, and the code used for these plots is available in a function called *circomap* [39]. All datasets are available at the NCI genomic data commons website [40]. Details on how the data was processed for the results shown in the manuscript are discussed in Additional file 1: Supplementary Note.
