# Supplementary material for: Pan-cancer identification of clinically relevant genomic subtypes using outcome-weighted integrative clustering
Source: Genome Med. 2020 Dec 3;12:110. doi: 10.1186/s13073-020-00804-8 (PMC7716509; doi:10.1186/s13073-020-00804-8)
Supplement: Supplementary file 1 — Additional file 1: Supplemental File containing all Supplemental Figures, Supplemental Tables, and Supplemental Note including additional details on the method and data analysis. Table S1. Supplementary Table summarizing input data of 18 cancer types across number of samples analyzed and total number of features that went into clustering for mutation, Copy Number (CN), Methylation, mRNA expression, miRNA and Protein. Table S2. Supplementary Table showing comparisons of survClust integrated solution versus unsupervised clustering results from published TCGA studies. Table S3. Summary of survival association for the survClust Copy Number solutions versus unsupervised TCGA solutions using the logrank statistic. Table S4. Summary of survival association for the survClust Methylation expression solution versus available TCGA solutions using the logrank statistic. Table S5. Summary of survival association for the survClust mRNA expression solution versus available TCGA solutions using the logrank statistic. Table S6. Summary of survival association for the survClust miRNA expression solution versus available TCGA solutions using the logrank statistic. Table S7. Summary of survival association for the survClust Protein expression solution vs available TCGA solutions using the logrank statistic. Table S8. Cross tabulation of the ovarian cancer copy number survClust class labels vs AKT gene Copy number changes. Figure S1. survClust simulation analysis. Figure S2. A second simulation example. Figure S3. survClust integrated solution of low grade glioma (LGG). Figure S4. survClust identifies tumor mutation burden (TMB) patterns across cancer types. Figure S5. CD8 expression stratified by the survClust mutation subclasses across various cancer types. Figure S6. survClus tidentifies copy number alteration patterns across cancer types, Global view of Copy Number Changes. Figure S7. survClust identifies FGA patterns across cancer types that associate with survival (Kaplan Meier cu [file 13073_2020_804_MOESM1_ESM.pdf]

Content		Page Number
**Table S1**	Summarizing input data of 18 cancer types across number of samples analyzed and total number of features that went into clustering for mutation, Copy Number (CN), Methylation, mRNA expression, miRNA and Protein	2
**Table S2**	Comparison of *survClust* **integrated** solution versus unsupervised clustering results from published TCGA studies	3
**Table S3**	summary of *survClust* **Copy Number** solution vs unsupervised TCGA solutions on logrank	4
**Table S4**	summary of *survClust* **Methylation expression** solution vs available TCGA solutions on logrank	5
**Table S5**	summary of *survClust* **mRNA expression** solution vs available TCGA solutions on logrank	6
**Table S6**	summary of *survClust* **miRNA expression** solution vs available TCGA solutions on logrank	7
**Table S7**	summary of *survClust* **Protein expression** solution vs available TCGA solutions on logrank	8
**Table S8**	Cross tabulation of OV copy number *survClust* labels vs Copy number change in *AKT* gene	9
**Fig S1**	Understanding survClust simulation and analysis	10
**Fig S2**	Another simulation example	11
**Fig S3**	*survClust* integrated solution of LGG	12
**Fig S4**	*survClust* identifies TMB patterns across cancer types	13
**Fig S5**	CD8 T-cell distribution of *survClust* mutation classes of various cancer types	14
**Fig S6**	*survClust* identifies FGA patterns across cancer types, Global view of Copy Number Change	15
**Fig S7**	*survClust* identifies FGA patterns across cancer types, Kaplan Meier curves	16
**Fig S8**	*survClust* integrated solution of various cancer types	17
**Supplementary Note**		18

Table S1 – Summarizing input data of 18 cancer types across number of samples analyzed and total number of features that went into clustering for mutation, Copy Number (CN), Methylation, mRNA expression, miRNA and Protein

cancer type	Total samples analyzed	mutation	CN	Methylation	mRNA	miRNA	RPPA
**BLCA**	411	8867	3353	9293	15695	553	189
**CESC**	293	5579	8471	9743	15771	565	193
**COAD**	432	7064	1909	9432	15331	494	NA
**ESCA**	185	1790	6508	9938	16997	535	193
**HNSC**	528	6304	7942	9662	15840	573	191
**KIRP**	289	1506	2274	7697	15675	502	190
**LGG**	512	665	4383	8866	15920	592	190
**LIHC**	372	2490	3719	9900	15106	550	190
**LUAD**	515	4803	2053	11546	15979	542	189
**LUSC**	503	4485	3650	9019	16227	543	189
**MESO**	86	43	3416	8506	15798	558	190
**OV**	537	2148	2432	7175	16847	531	189
**PAAD**	184	3494	4521	9061	16193	565	190
**SARC**	261	5868	4024	8840	15574	463	193
**STAD**	423	5595	4180	10583	15416	510	193
**UCEC**	542	9175	2320	8650	16811	540	189
**UCS**	56	1149	3706	8288	16194	633	193
**UVM**	80	87	749	6595	14626	557	NA

Table S2 – Comparison of *survClust* **integrated** solution versus unsupervised clustering results from published TCGA studies. The logrank test statistics (cross-validated) for survival association of the subtypes are reported.

**Cancer Type**	**survClust** Logrank statistic	**P-value**	**Unsupervised clustering** Logrank	**P-value**	**Algorithm**
Bladder	22.65	4.78E-05	4.8	1.87E-01	COCA
Cervical	12.47	1.42E-02	NA	NA	--
Colorectal	5.2	2.67E-01	0.61	8.95E-01	PARADIGM
Esophageal	1.73	6.30E-01	1.08	8.97E-01	iCluster
Head and neck	24.41	6.60E-05	8.73	3.31E-02	Manual
Kidney papillary	129.79	P<1E-05	79.09	P<1E-05	COCA
Low grade glioma	288.68	P<1E-05	253.45	P<1E-05	COCA
Liver	21.56	2.45E-04	1.71	4.24E-01	iCluster
Lung adeno	15.13	1.93E-02	9.09	1.06E-01	iCluster
Lung squamous	5.06	2.81E-01	0.81	8.48E-01	iCluster
Mesothelioma	7.86	1.96E-02	NA	NA	--
Ovarian	7.34	2.55E-02	4.93	1.77E-01	
Pancreas	14.7	6.43E-04	NA	NA	--
Soft tissue sarcoma	17.94	1.27E-03	NA	NA	--
Stomach	5.2	7.44E-02	2.65	6.18E-01	Manual
Endometrial	45.65	6.72E-10	36.21	6.76E-08	Manual
Uterine	0.92	6.33E-01	0.71	3.98E-01	
Uveal melanoma	16.16	1.05E-03	NA	NA	--

Table S3 – summary of *survClust* **Copy Number** solution vs unsupervised TCGA solutions on logrank (columns 1 and 2). Total number of samples analyzed via *survClust* (column 3), and total samples analyzed via unsupervised clustering (column 4)

Cancer type	survClust logrank	unsup logrank	survClust (N)	unsup(N)
**BLCA**	3.45	NA	405	NA
**CESC**	0.76	NA	280	NA
**COAD**	5.9	NA	409	NA
**ESCA**	0.96	NA	182	NA
**HNSC**	18.64	9.86	517	279
**KIRP**	57.73	46.93	281	161
**LGG**	246.74	NA	507	NA
**LIHC**	51.95	3.16	362	193
**LUAD**	5.96	NA	496	NA
**LUSC**	3.1	NA	486	NA
**MESO**	3.45	NA	86	NA
**OV**	27.13	NA	558	NA
**PAAD**	8.96	NA	182	NA
**READ**	3.92	NA	148	NA
**SARC**	12.59	NA	252	NA
**STAD**	2.18	NA	419	NA
**UCEC**	35.54	19.64	518	537
**UCS**	NA	NA	NA	NA
**UVM**	16.53	NA	80	NA

Table S4 – summary of *survClust* **Methylation expression** solution vs available TCGA solutions on logrank (columns 1 and 2). Total number of samples analyzed via *survClust* (column 3), and total samples analyzed via TCGA (column 4)

Cancer type	survClust logrank	TCGA logrank	survClust(N)	TCGA(N)
**BLCA**	9.89	NA	411	NA
**CESC**	3.83	NA	292	NA
**COAD**	2.92	NA	424	NA
**ESCA**	9.7	NA	183	NA
**HNSC**	20.68	6.39	523	279
**KIRP**	98.05	74.24	284	161
**LGG**	113.55	247.85	510	512
**LIHC**	4.27	7.15	369	193
**LUAD**	0.64	4.51	508	228
**LUSC**	3.34	NA	488	NA
**MESO**	0.17	NA	86	NA
**OV**	4.21	NA	575	NA
**PAAD**	15.79	NA	182	NA
**READ**	0.69	NA	148	NA
**SARC**	7.2	NA	257	NA
**STAD**	5.07	NA	421	NA
**UCEC**	28.76	NA	526	NA
**UCS**	0.13	NA	56	NA
**UVM**	22.14	NA	80	NA

Table S5 – summary of *survClust* **mRNA expression** solution vs available TCGA solutions on logrank (columns 1 and 2). Total number of samples analyzed via *survClust* (column 3), and total samples analyzed via TCGA (column 4)

Cancer type	survClust logrank	TCGA logrank	survClust(N)	TCGA(N)
**BLCA**	18.51	4.8	407	129
**CESC**	9.9	NA	290	NA
**COAD**	NA	NA	NA	NA
**ESCA**	1.9	NA	184	NA
**HNSC**	25	8.73	520	279
**KIRP**	93.19	20.68	288	161
**LGG**	139.33	149.71	512	512
**LIHC**	26.87	4.54	366	193
**LUAD**	17.96	NA	511	NA
**LUSC**	5.33	0.81	500	178
**MESO**	21.24	NA	86	NA
**OV**	5.15	4.93	303	489
**PAAD**	14.9	NA	177	NA
**READ**	0.87	NA	153	NA
**SARC**	8.62	NA	259	NA
**STAD**	11.66	NA	395	NA
**UCEC**	26.06	11.26	531	537
**UCS**	2.12	0.71	56	56
**UVM**	13.95	NA	80	NA

Table S6 – summary of *survClust* **miRNA expression** solution vs available TCGA solutions on logrank (columns 1 and 2). Total number of samples analyzed via *survClust* (column 3), and total samples analyzed via TCGA (column 4)

Cancer type	survClust logrank	TCGA logrank	survClust(N)	TCGA(N)
**BLCA**	17.95	NA	408	NA
**CESC**	3.31	NA	292	NA
**COAD**	4.81	NA	402	NA
**ESCA**	4.55	NA	182	NA
**HNSC**	12.89	2.52	519	279
**KIRP**	26.95	8.56	284	161
**LGG**	84.91	NA	506	NA
**LIHC**	28.21	6.99	364	193
**LUAD**	7.28	NA	504	NA
**LUSC**	7.85	NA	466	NA
**MESO**	21.3	NA	86	NA
**OV**	12.47	NA	475	NA
**PAAD**	12.43	NA	176	NA
**READ**	0.13	NA	146	NA
**SARC**	11.36	NA	256	NA
**STAD**	8.76	NA	414	NA
**UCEC**	0.86	NA	522	NA
**UCS**	0.96	NA	55	NA
**UVM**	12.93	NA	80	NA

Table S7– summary of *survClust* **Protein expression solution** vs available TCGA solutions on logrank (columns 1 and 2). Total number of samples analyzed via *survClust* (column 3), and total samples analyzed via TCGA (column 4)

Cancer type	survClust logrank	TCGA logrank	survClust(N)	TCGA(N)
**BLCA**	11.98	NA	343	NA
**CESC**	9.74	NA	164	NA
**COAD**	NA	NA	NA	NA
**ESCA**	1.48	NA	126	NA
**HNSC**	17.77	5.96	212	279
**KIRP**	49.42	1.41	215	161
**LGG**	53.74	14.06	425	512
**LIHC**	4.16	0.25	182	193
**LUAD**	2.26	NA	362	NA
**LUSC**	7.8	NA	328	NA
**MESO**	9.18	NA	63	NA
**OV**	12.62	NA	422	NA
**PAAD**	0.39	NA	123	NA
**READ**	NA	NA	NA	NA
**SARC**	18.89	NA	223	NA
**STAD**	NA	NA	NA	NA
**UCEC**	17.86	NA	439	NA
**UCS**	0.26	NA	47	NA
**UVM**	NA	NA	NA	NA

Table S8 – Cross tabulation of OV copy number *survClust* labels vs Copy number change in *AKT* gene

AKT2	survClust CN labels c1	c2	c3	c4	c5	c6
-2	0	0	1	1	0	0
-1	26	14	84	30	3	31
0	76	46	3	43	1	4
1	49	42	1	29	31	10
2	4	7	0	3	18	1

Fig. S1

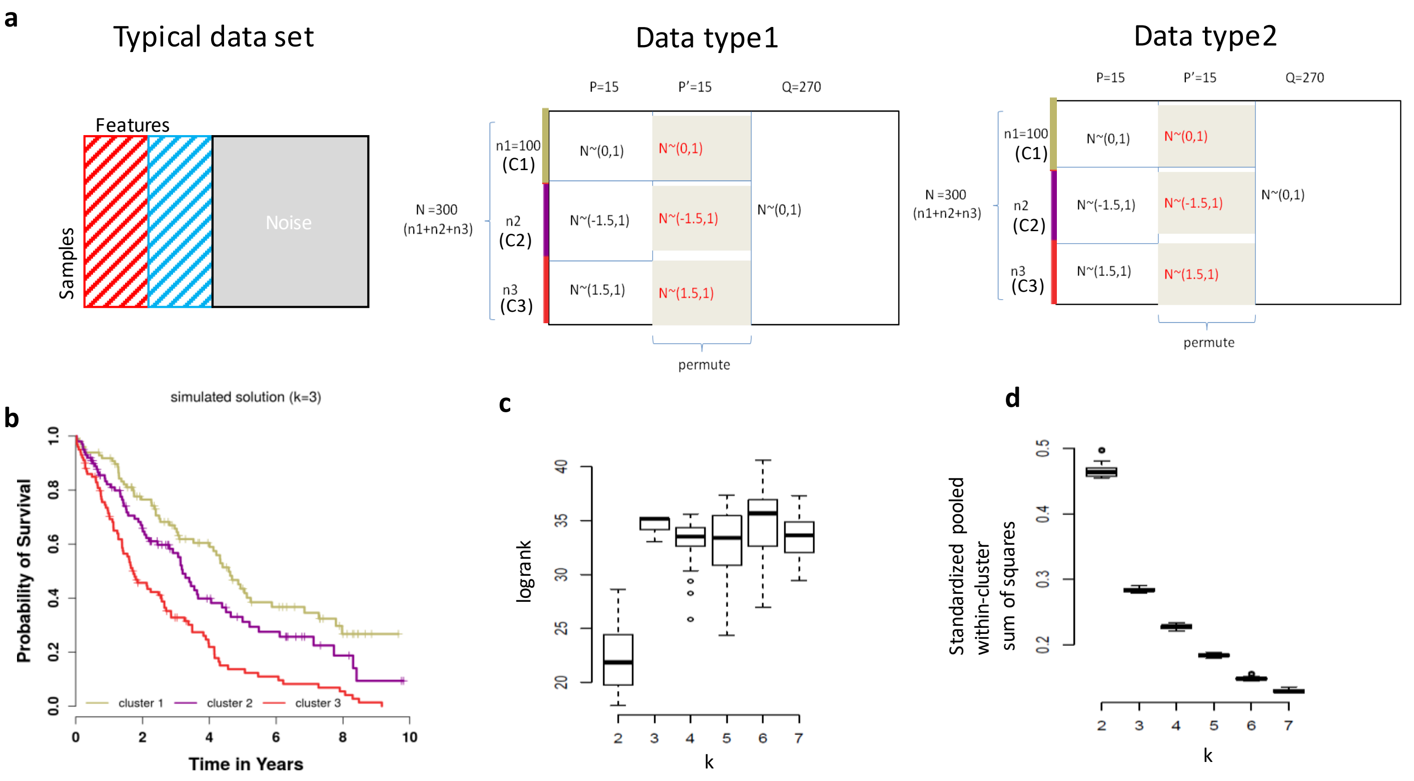

e

survClust labels	c1	c2	c3
Simulated labels			
Cluster 1	100	0	0
Cluster 2	0	100	0
Cluster 3	0	0	100

Fig. S1. Simulation analysis.
(a) A typical data set with survival related features, survival unrelated features and noise. **Data Type 1** – 15 out of 300 features have a distinct structure along with survival association, next 15 were simulated in the same manner but permutated, and remaining features were noise. **Data Type 2 –** Same as Data type 1.

(b) Simulated 3-class survival with median survival time as 4,3, and 2 years in clusters 1,2 and 3 respectively.

(c) Boxplot of logrank of 50 rounds of cross validation with *survClust.*

(d) Boxplot of standardized pooled within-cluster sum of squares of 50 rounds of cross validation with *survClust.*

(e) Cross tabulation with *survClust* labels with respect to simulated class labels or truth.

Fig. S2

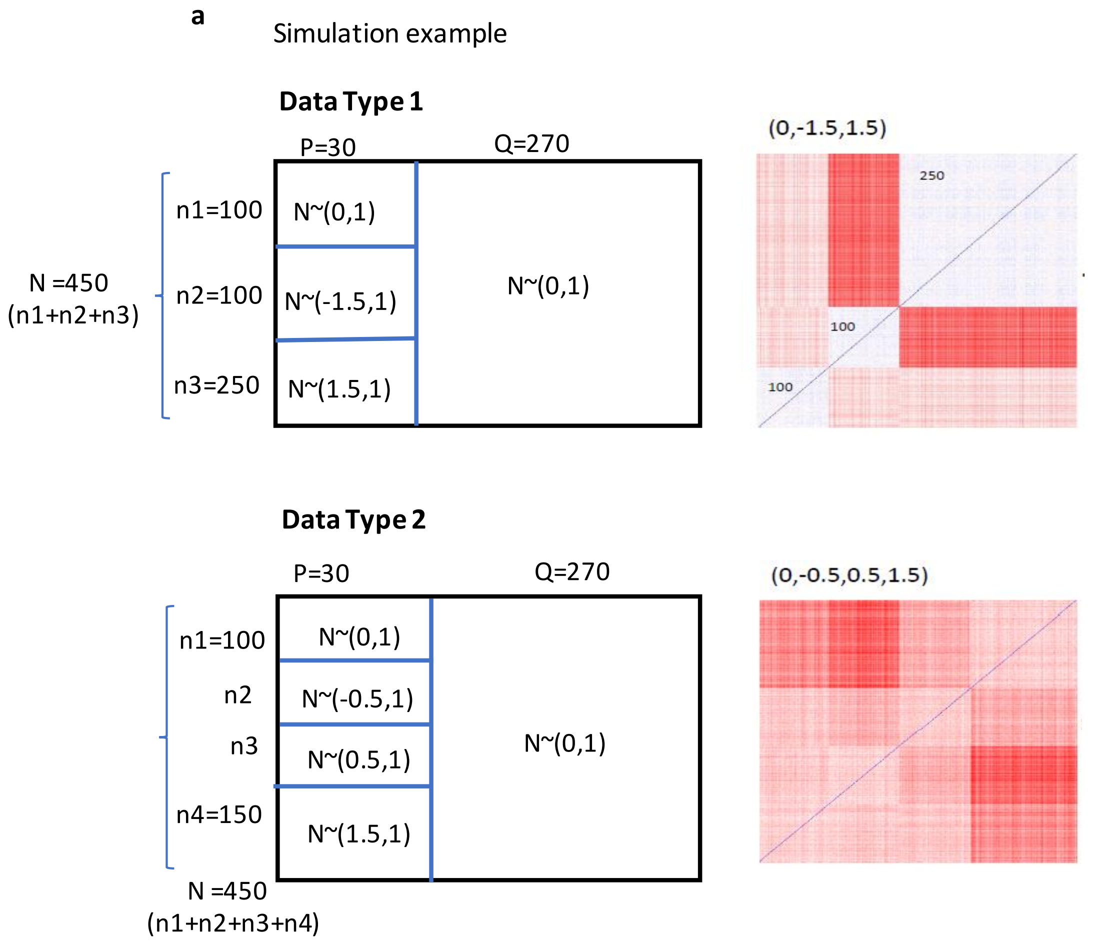

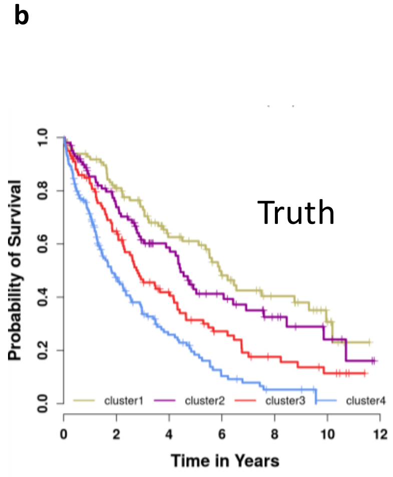

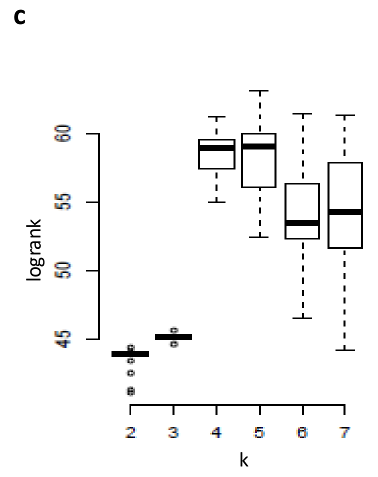

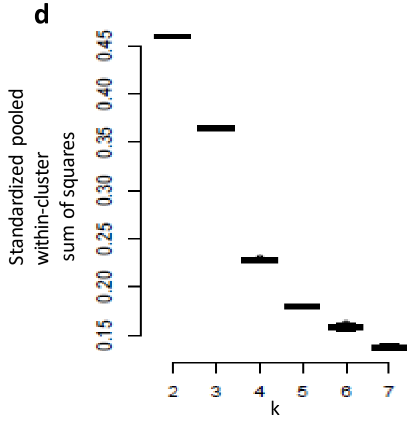

e

survClust labels	c1	c2	c3	c4
Simulated labels				
Cluster 1	100	0	0	0
Cluster 2	0	100	0	0
Cluster 3	0	0	100	0
Cluster 4	0	0	0	150

Fig. S2. Second simulation analysis.
(a) Simulated structures of two data types. **Data type 1** – strong clusters, weak survival association. Plot on the right is the weighted distance matrix of data type 1. **Data type 2** – weak clusters, strong cluster association. Plot on the right is the weighted distance matrix of data type 2.

(b) Simulated 4-class survival with median survival time as 5,4,3, and 2 years in clusters 1,2,3 and 4 respectively.

(c) Boxplot of logrank of 50 rounds of cross validation with *survClust.*

(d) Boxplot of standardized pooled within-cluster sum of squares of 50 rounds of cross validation with *survClust.*

(e) Cross tabulation with *survClust* labels with respect to simulated class labels or truth.

Fig. S3

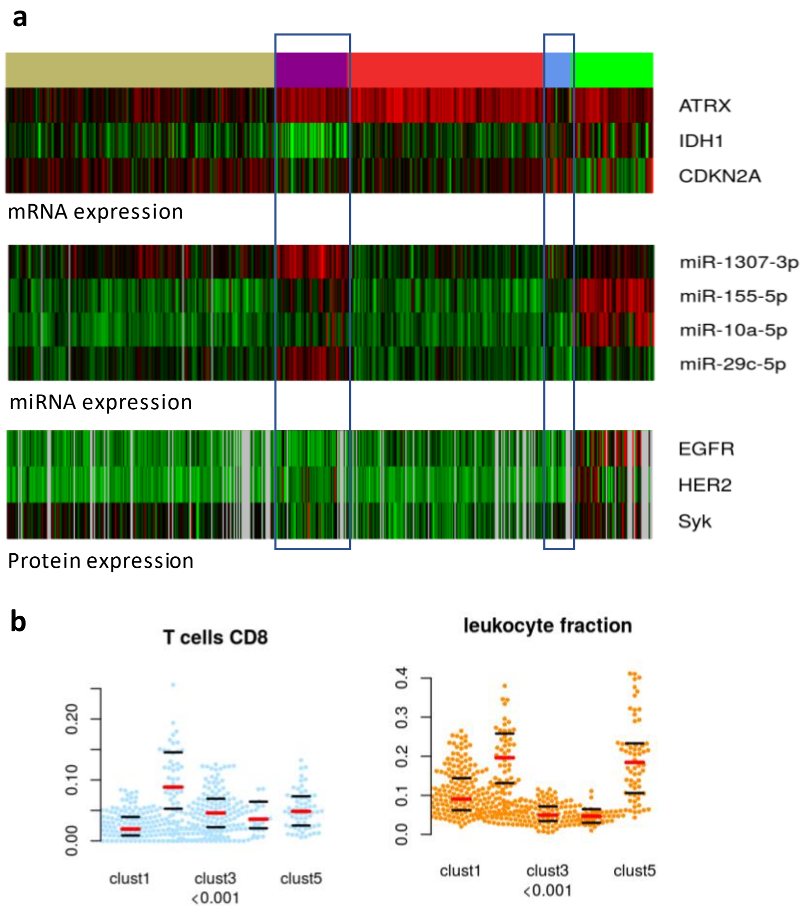

Fig. S3: *survClust* integrated solution of LGG

(a) Selected gene, miRNA and protein expression with respect to 5-class LGG integrated *survClust* labels. **(b)** Beeswarm plots summarizing infiltration levels (y-axis) of CD8 T-cells and leukocyte fraction across the five groups (x-axis). Red line depicts the median, and top and bottom black bars represent 25th and 75th percentile respectively.

Fig. S4

Mutation - Yes
Missing
HPV negative
HPV Positive
Yes
No

c1 c3 c5
c2 c4 c6

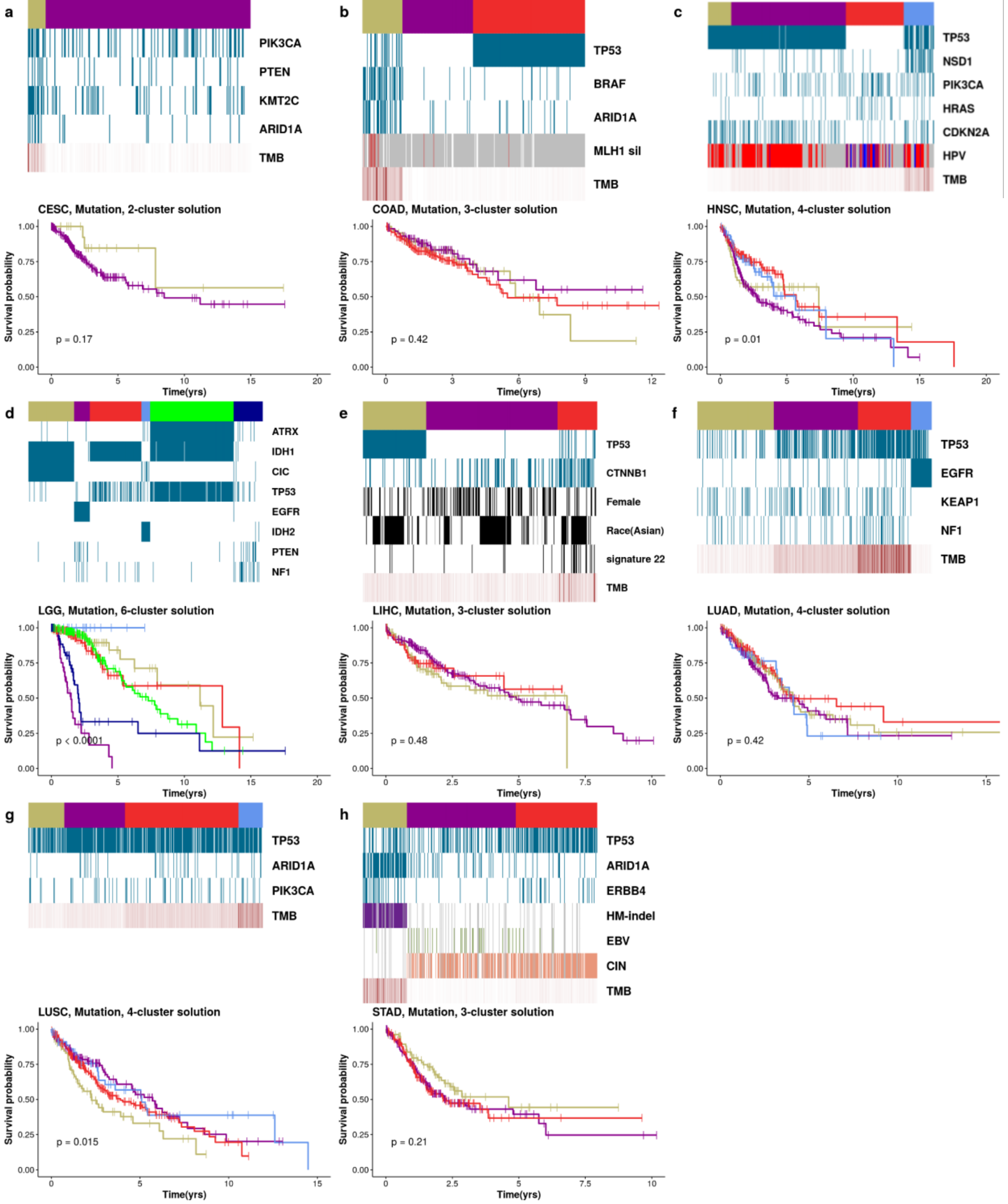

Fig. S4: *survClust* identifies TMB patterns across cancer types

Each sub figure contains *panelmap* summarizing differentiating molecular and clinical characteristics for *k* cluster *survClust* solution labels on mutation data alone, followed by Kaplan-Meier curves for each group.
(a)CESC **(b)**COAD **(c)**HNSC **(d)**LGG **(e)**LIHC **(f)**LUAD **(g)**LUSC **(h)**STAD

Fig. S5

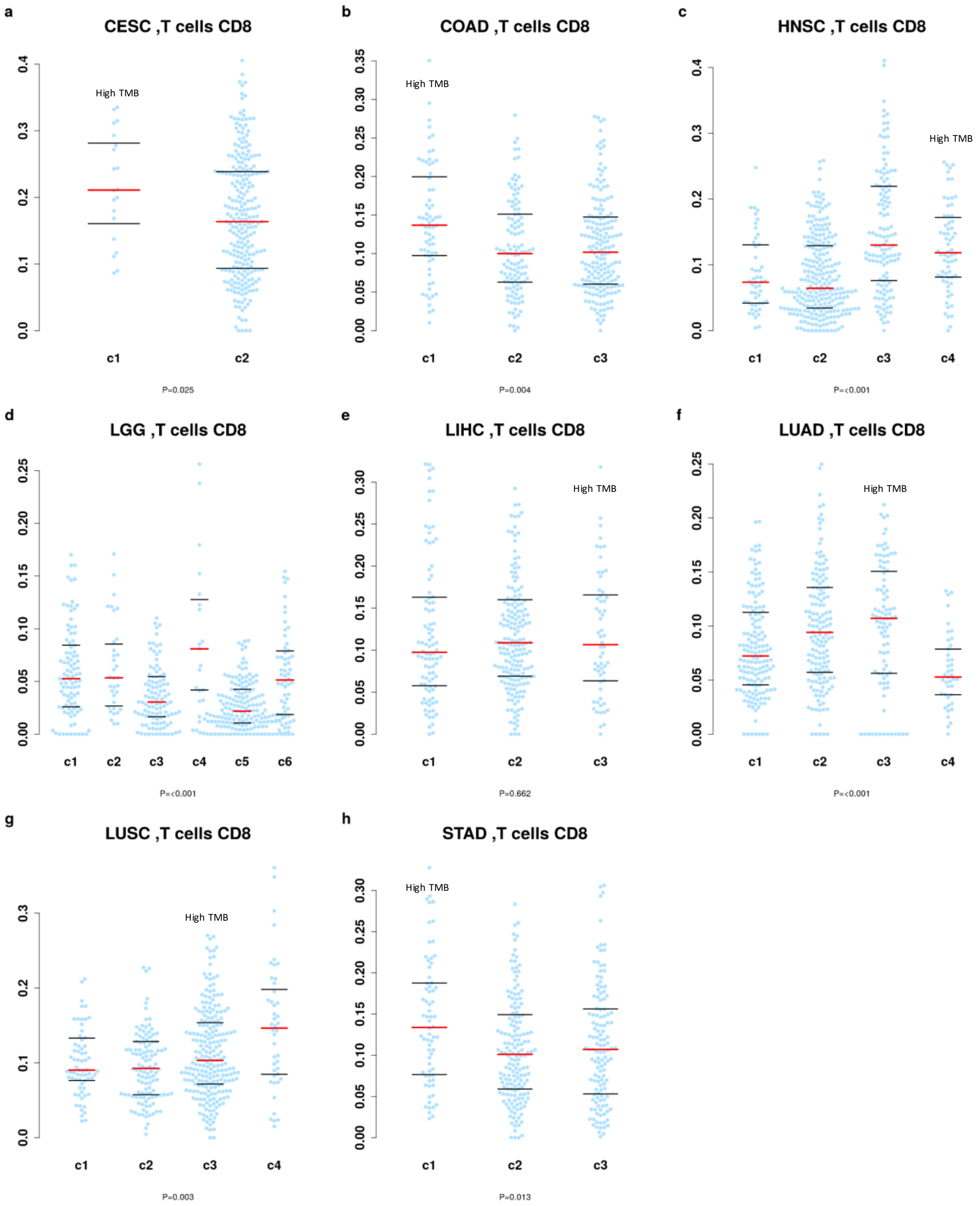

Fig. S5: CD8 T-cell distribution of *survClust* mutation classes of various cancer types

Boxplot summarizing CD8 T-cell expression (y-axis) across survClust class labels (x-axis). Red line depicts the median, and top and bottom black bars represent 25th and 75th percentile respectively. Significance from association test is shown at the bottom of each plot.
(a)CESC **(b)**COAD **(c)**HNSC **(d)**LGG **(e)**LIHC **(f)**LUAD **(g)**LUSC **(h)**STAD

Fig. S6

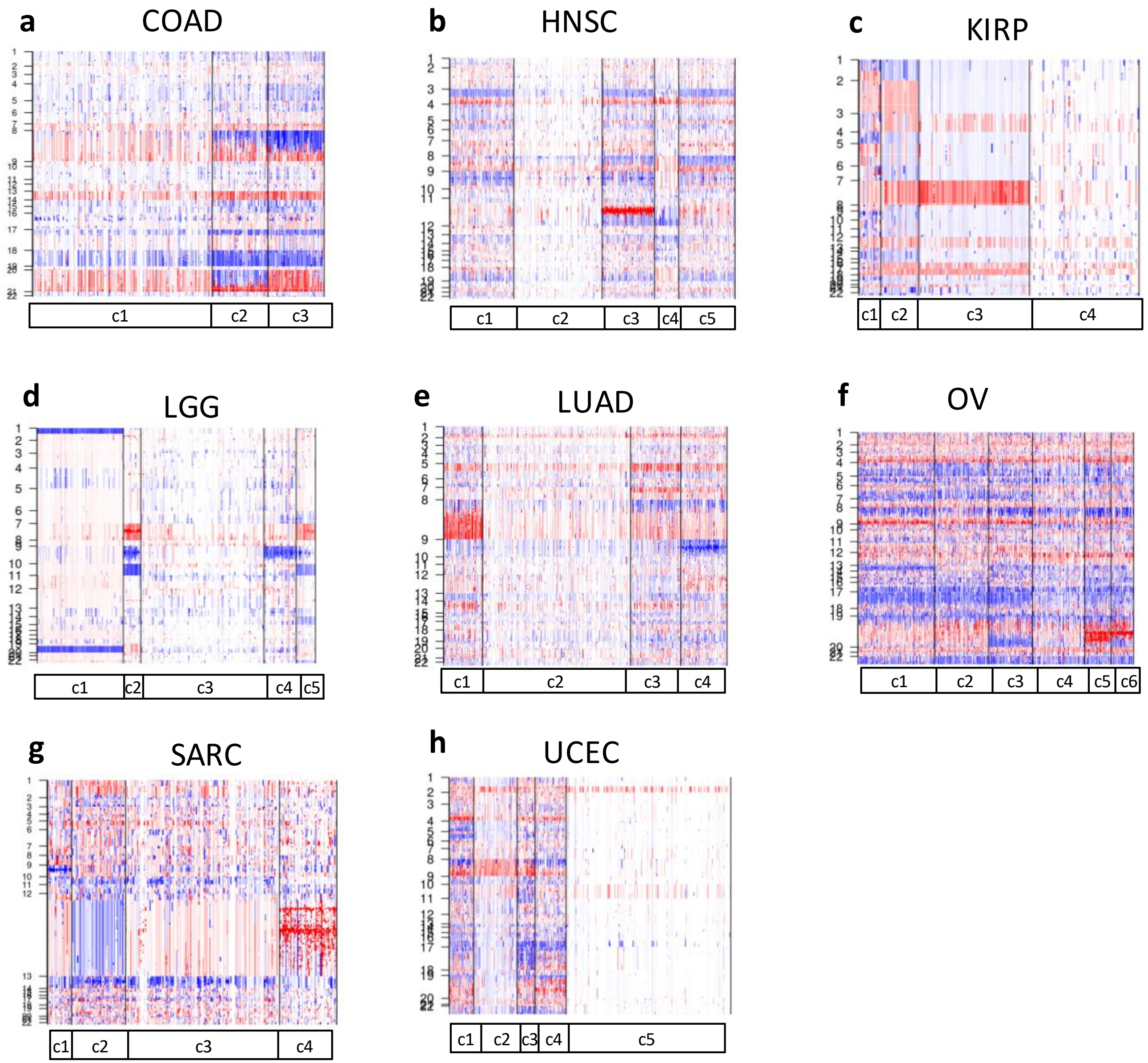

Fig. S6: *survClust* identifies FGA patterns across cancer types: Global view of Copy Number Change
Figures a-i represent a global view of copy number changes (Chromosome 1-22) in cluster labels identified by survClust run on segmented copy number data. **(a)**COAD **(b)**HNSC **(c)**KIRP **(d)**LGG **(e)**LUAD **(f)**OV **(g)**SARC **(h)**UCEC.

Fig. S7

Fig. S7: *survClust* identifies FGA patterns across cancer types: Kaplan-Meier Curves

Kaplan-Meier curves for each group obtained by *survClust* run on segmented copy number data for various cancer types.
(a)COAD **(b)**HNSC **(c)**KIRP **(d)**LGG **(e)**LIHC **(f)**LUAD **(g)**OV **(h)**SARC **(i)**UCEC

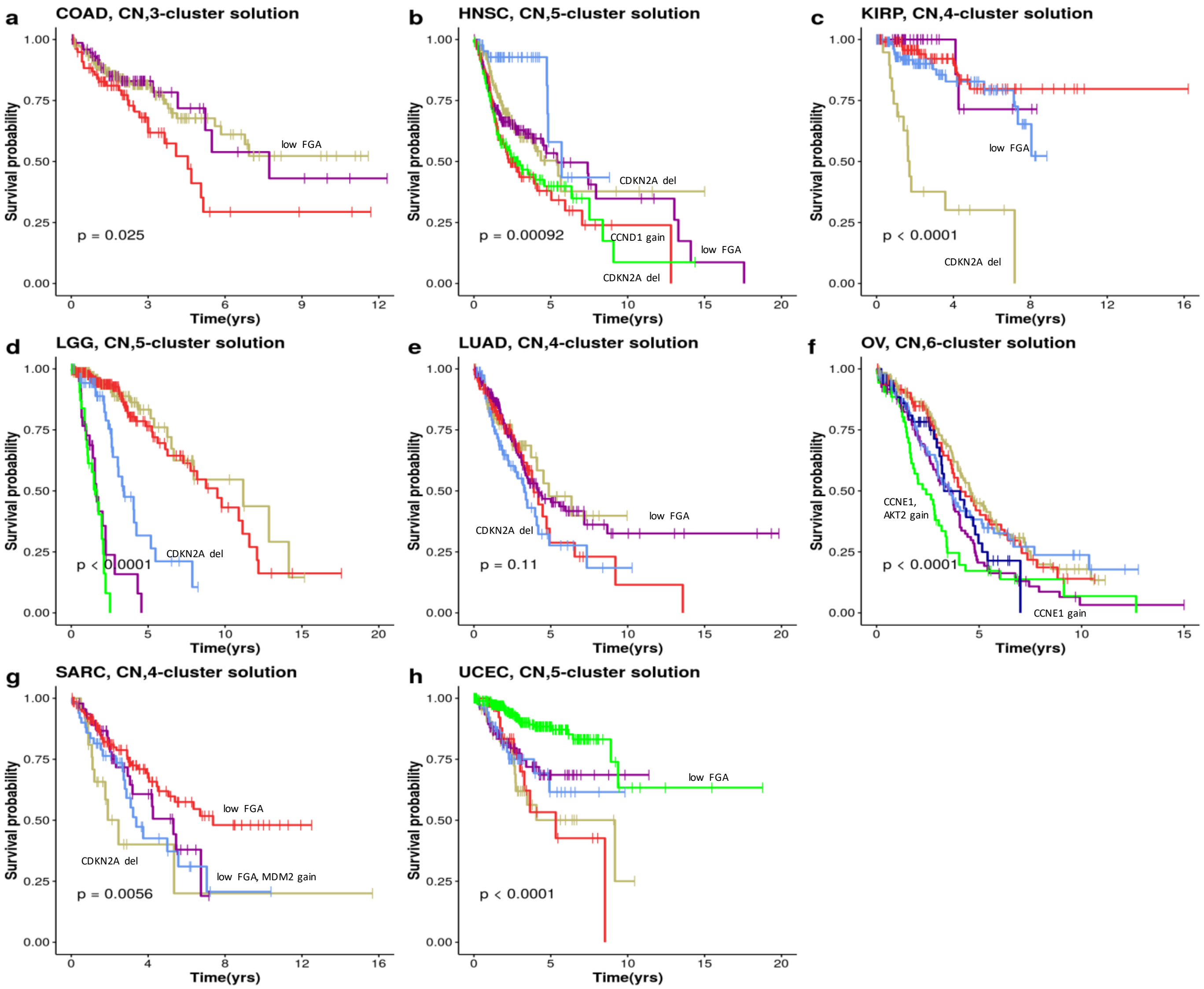

CCNE! gain

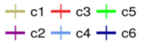

Fig. S8

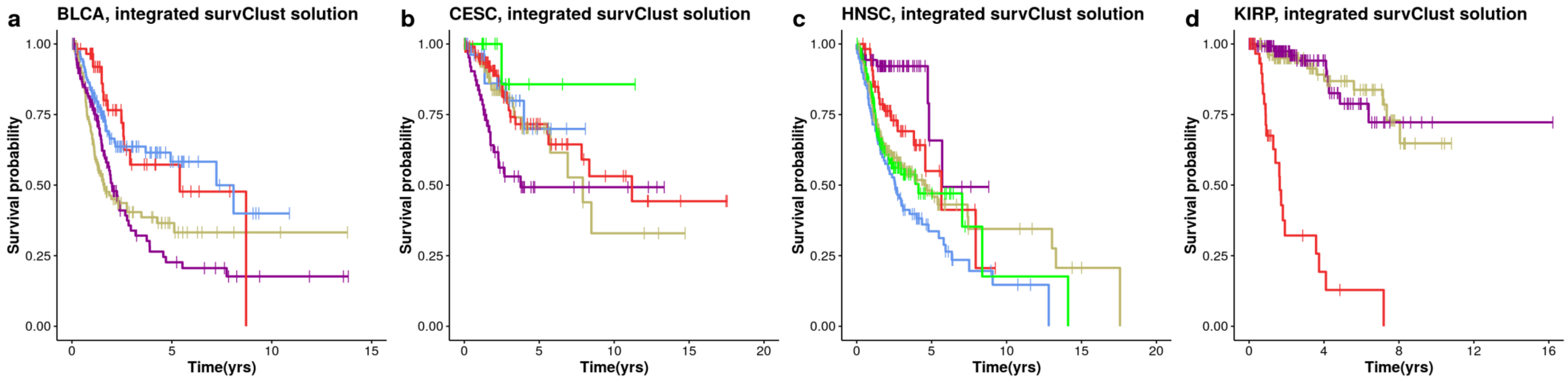

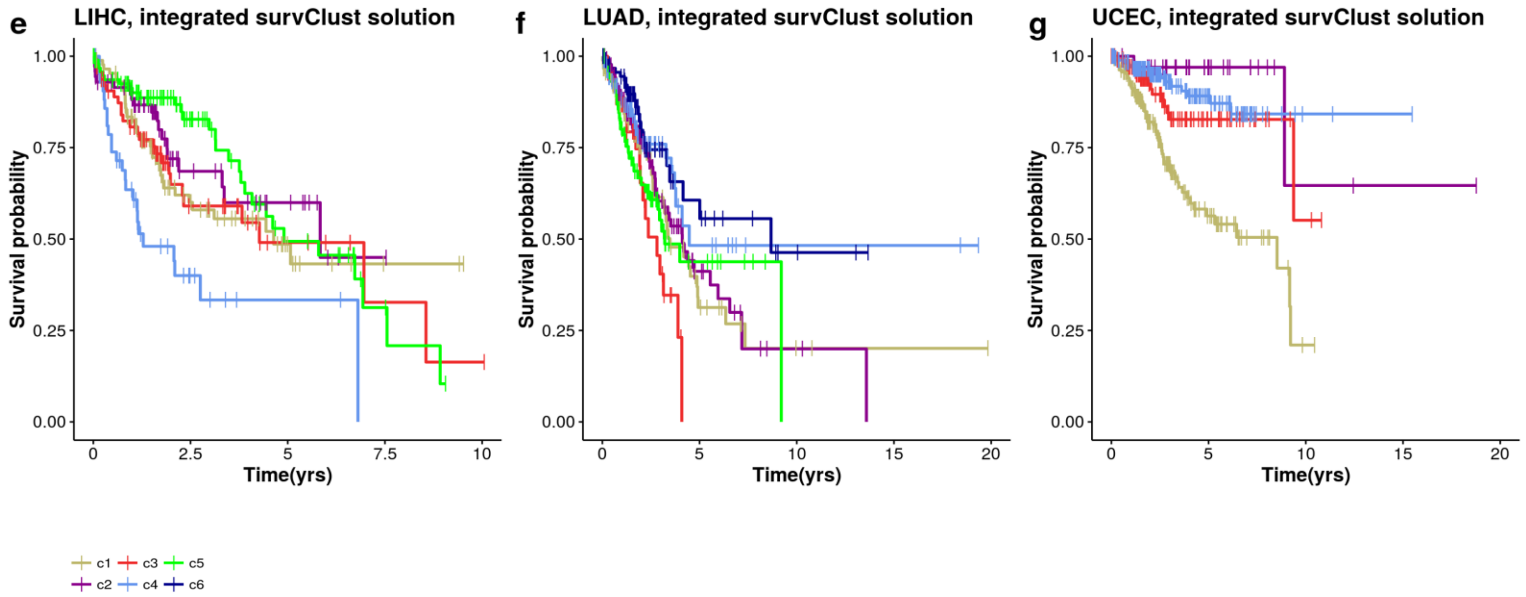

Fig. S8: *survClust* integrated solution of various cancer types

Kaplan-Meier curves for solutions obtained by *survClust* run on integrating six platforms – DNA methylation, Copy Number, mRNA expression, miRNA expression, protein expression and mutation data.
(a)BLCA **(b)**CESC**(c)**HNSC **(d)**LIHC**(e)**KIRP**(f)**LUAD **(g)**CESC

Supplementary Notes

1. Data Pre-Processing

Data types may consist of continuous (gene expression, copy number log-ratio, DNA methylation, miRNA, protein expression) or binary (mutation status) data. Each data type is pre-processed by normalization and standardized as follows –

$$X_{standardized} = \frac{\boldsymbol{X} - \overline{\boldsymbol{X}}}{\boldsymbol{sd}(\boldsymbol{X})}$$

where X is a data type.

Copy Number data was segmented using CBS[1] and reduced to non-redundant regions of alterations using the CNregion function in iClusterPlus[2] with default epsilon of 0.001, keeping in mind that the total numbers of features don't exceed 10,000. For DNA methylation, mRNA expression and miRNA expression, if a certain feature had more than 20% missing data, that feature was removed and remaining were used for analysis. For mRNA expression, we further removed genes having a mean expression lower than the threshold of mean expression of lower 10% quantile. Similarly, methylation probes with mean beta values < 0.1 and > 0.9 were discarded. Genes harboring mutants in less than 1% of the samples were removed. Missing data was imputed using KNN imputation[3]. Data for each cancer type and assay was extracted from pan-cancer study[4]. A summary of features and samples across all cancer types analyzed is shown in **Additional file 1: Table S1**. Mutation signature analysis was run on the resulting maf file using (https://github.com/mskcc/mutation-signatures)[5]. Blood biomarker data was derived from CIBERSORT[6].

2. Running survClust

survClust was run on all cancer types reported in **Additional file 1: Table S1**. Results are summarized according to each cancer type. Each cancer type was run for each of the 6 platforms - somatic mutation, DNA copy number, DNA methylation, mRNA expression, miRNA expression and RPPA data, and integrating all six.
Final results were compiled after running *cv.survclust* for 5 folds for 50 rounds of cross validation. Optimal k was chosen after assessing cross-validated logrank statistic and standardized pooled within cluster sum of squares. Overfitting was avoided by omitting cluster solution with less than 5 samples. Note that, one can considerably reduce the time to run survClust by reducing the feature space.

3. Simulation details

We present here two examples of simulations to go over the conceptualization of survClust.

3.1 Simulation 1

We simulated a data matrix with 300 samples and 300 features. Out of which 15 features, f, were coded for survival relevant information for 100 samples each in cluster1,2 and 3 respectively -

$$\boldsymbol{C1}_{(\boldsymbol{f=15})} \sim N(0,1),\ \ \boldsymbol{C2}_{(\boldsymbol{f=15})} \sim N(-1.5,1), \boldsymbol{C3}_{(\boldsymbol{f=15})} \sim N(1.5,1) \qquad (\mathbf{1})$$

Next 15 were coded for survival unrelated features and formed the competing cluster. Survival association was perturbed by permuting the samples, which dropped the associated survival information within features but retained the molecular distinction between clusters.

$$\boldsymbol{C1}_{(\boldsymbol{f_{permute}=15})} \sim N(0,1),\ \ \boldsymbol{C2}_{(\boldsymbol{f_{permute}=15})} \sim N(-1.5,1),\ \boldsymbol{C3}_{(\boldsymbol{f_{permute}=15})} \sim N(1.5,1)$$

$$(\mathbf{1.1})$$

And remaining 270 features were simulated as noise –

$$\boldsymbol{C1}_{(\boldsymbol{f=270})} \sim N(0,1),\ \ \boldsymbol{C2}_{(\boldsymbol{f=270})} \sim N(0,1), \boldsymbol{C3}_{(\boldsymbol{f=270})} \sim N(0,1) \qquad (\mathbf{1})$$

Survival correlation was imposed by simulating C1, C2 and C3, to have a median survival time of 4, 3 and 2 years respectively. The simulated survival data observed right censoring, with each individual having failure time as T and censoring time as C, and time-to-event data as follows –

$$\begin{aligned} Y &= \min(T, C) = T \perp C \\ \delta &= \begin{cases} 1, if\ T = Y \\ 0\ \ otherwise \end{cases} \\ T &\sim \exp(\lambda)\ , C \sim U(a, b) \end{aligned} \qquad (\mathbf{2})$$

Where, λ=median survival rate, such as, a median survival rate of 4 years is - log(2)/4. *a,b* are minimum and maximum follow up times.
We created two such data types to showcase integration capabilities of survClust (**Additional file 1: Fig. S1**). We ran survClust and cross-validated runs for k=2-7 for 50 rounds. Optimum k is chosen where logrank is maximized and standardized pooled within-cluster sum of squares is minimized. Results are shown in Figure 1.

3.2 Simulation 2

We show another simulation example, where we simulate two data types where the truth is a 4-class solution shown in **Additional file 1: Fig. S2**, such that:

$$\textbf{Data type 1}:\ \boldsymbol{C1}_{(\boldsymbol{f=15,N=100})} \sim N(0,1),\ \ \boldsymbol{C2}_{(\boldsymbol{f=15,N=100})} \sim N(-1.5,1),$$

$$\boldsymbol{C3}_{(\boldsymbol{f=15,N=250})} \sim N(1.5,1)$$

$$\textbf{Data type 2}:\ \boldsymbol{C1}_{(\boldsymbol{f=15,N=100})} \sim N(1.5,1),\ \ \boldsymbol{C2}_{(\boldsymbol{f=15,N=100})} \sim N(0.5,1),$$

$$\boldsymbol{C3}_{(\boldsymbol{f=15,N=100})} \sim N(0,1), \boldsymbol{C4}_{(\boldsymbol{f=15,N=150})} \sim N(-0.5,1)$$

Data type 1 shows strong molecular association, whereas data type 2 shows strong survival association and weak molecular association.

Survival correlation was imposed by simulating C1, C2, C3 and C4 with median survival time of 5, 4, 3 and 2 years respectively, see (2). The goal here was to see if survClust can identify 4 distinct survival groups when a data type has a strong 3-class molecular structure (as shown in Data type1). Results shown in **Additional file 1: Fig. S2**.

4. Centroid Re-labelling

We performed cross validation to avoid over fitting and to arrive at coherent survClust results. Each fold predicts test labels according to its training set, and at the end of cross validation we have prediction of each sample to a class. However, the class labels are meaningless across folds and one needs to be careful when aggregating labels to get a full solution. We define, centroid relabeling method to solve this problem.

Let N be total number of samples to classify. We perform a F fold cross validation, where each fold predicts a test set, such that,

$\sum_{f=1}^{F} test\ set_f = N.$

Assume we have the following class label prediction for test set for k-class solutions across F folds.

$test\ set_1 = a_{11}, a_{12}, a_{13}, a_{24}, a_{35}, a_{26} .. a_{ki};$; $test\ set_2 = a_{11}, a_{12}, a_{13}, a_{24}, a_{35}, a_{26} .. a_{ki};$ $\ldots\ test\ set_f = a_{11}, a_{12}, a_{13}, a_{24}, a_{35}, a_{26} .. a_{ki}$

Where a_{ki} = i^{th} sample belonging to k^{th} class in a test set fold f. One can clearly see that k labels across f folds are meaningless and simply group alike samples together and unlike samples separately in different clusters. To classify all N samples, we need to consolidate the labels across F folds.

Lets consider the following example where we know the true class of each sample -

**Sample**	**Class**
sample1	1
sample2	2
sample3	3
sample4	1
sample5	2
sample6	3
sample7	1
sample8	2
sample9	3

Now we perform 3-fold cross validation, using 2/3rd of the samples (n=6) to predict the remaining 1/3rd (n=3) samples in each fold.

test set prediction- Fold 1		Fold 2		Fold 3	
sample4	class1	sample5	class1	sample6	class1
sample2	class2	sample3	class2	sample1	class2
sample9	class3	sample7	class3	sample8	class3

As seen, these class labels are meaningless, but they group alike samples together and unlike samples separately. For example, Sample4, Sample5 and Sample6 are grouped in class1 across folds, but we know that their true label is class1, class2 and class3 respectively. We arrive at consolidated labels as follows-

Step 1- Calculate centroids of each $k(k = 3)$ class in each fold $f(f = 3)$.

$$Fold\ 1\ centroids -\ o_{11}, o_{21}, o_{31}$$

$$Fold\ 2\ centroids -\ o_{12}, o_{22}, o_{32}$$

$$Fold\ 3\ centroids -\ o_{13}, o_{23}, o_{33}$$

Where, $o_{kf}; k = k\ class$ and $f = f\ fold$

Where, $o_{kf} = \frac{\sum_k a_{k1}, + a_{k2} + a_{k3} + \cdots + a_{ki}}{i}$; centroid calculation for f^{th} fold and k^{th} class and i samples belonging to that k class.

Step 2- Run kmeans on the centroids vectors

$$kmeans(\ o_{11}, o_{12}, o_{13}, o_{21}, o_{22}, o_{23}, o_{31}, o_{32}, o_{33})$$

Step 3 – Get kmeans class labels on the centroids, and relabel each sample accordingly.

Kmeans output	Centroids
k=1	o_{11}, o_{32}, o_{23}
k=2	$o_{21}\ , o_{12}, o_{33}$
k=3	$o_{31}, o_{22,}\ o_{13}$

Re-assigned class labels -

Fold1		Fold2		Fold3	
Sample4	class1	Sample5	Class2	Sample6	Class3
Sample2	class2	Sample3	Class3	Sample1	Class1
Sample9	class3	Sample7	Class1	Sample8	Class2

This same strategy is used to relabel class membership of samples across rounds of cross validation, where each round has predicted class labels after each round of cross validation.

For a particular k each round r $(1,2,3 \ldots, R)$ of cross validation, we have relabeled class labels as described above, as $cv\ labels_1, cv\ labels_2, \ldots\ cv\ labels_R$.

Consolidate labels across all R rounds to arrive at the final solution as follows.

Compute centroids for each k class in each round R (same as Step1).

$r_1\, centroids -\ o_{11}, o_{21}, o_{31}, \ldots\ o_{k1}; r_2\ centroids -\ o_{12}, o_{22}, o_{32}, \ldots\ o_{k2}; r_R\ centroids -\ o_{1R}, o_{2R}, o_{3R}, \ldots\ o_{kR}$

Proceed to Step 2 and Step 3 to get relabeled class labels across each r round. Now the cluster membership has same meaning across all rounds of cross validation. A final class label is picked that is seen maximum number of times over R rounds.

References

1. Olshen, A. B., Venkatraman, E., Lucito, R. & Wigler, M. J. B. Circular binary segmentation for the analysis of array-based DNA copy number data. 5, 557-572 (2004).

2. Mo, Qianxing, and Ronglai Shen.iClusterPlus: integrative clustering of multiple genomic data sets.(2013).

3. Trevor Hastie, Robert Tibshirani, Balasubramanian Narasimhan and Gilbert Chu (2017). impute: impute: Imputation for microarray data. Rpackage version 1.50.1.

4. Hoadley, K. A. *et al.* Cell-of-origin patterns dominate the molecular classification of 10,000 tumors from 33 types of cancer. 173, 291-304. e296 (2018).

5. Alexandrov, L. B. *et al.* Signatures of mutational processes in human cancer. 500, 415 (2013)

6. Newman, A. M. *et al.* Robust enumeration of cell subsets from tissue expression profiles. 12, 453 (2015).
